# Molecular design of the γδT cell receptor ectodomain encodes biologically fit ligand recognition in the absence of mechanosensing

**DOI:** 10.1073/pnas.2023050118

**Published:** 2021-06-25

**Authors:** Robert J. Mallis, Jonathan S. Duke-Cohan, Dibyendu Kumar Das, Aoi Akitsu, Adrienne M. Luoma, Debasis Banik, Hannah M. Stephens, Paul W. Tetteh, Caitlin D. Castro, Sophie Krahnke, Rebecca E. Hussey, Brian Lawney, Kristine N. Brazin, Pedro A. Reche, Wonmuk Hwang, Erin J. Adams, Matthew J. Lang, Ellis L. Reinherz

**Affiliations:** ^a^Laboratory of Immunobiology, Dana-Farber Cancer Institute, Boston, MA 02115;; ^b^Department of Biological Chemistry and Molecular Pharmacology, Harvard Medical School, Boston, MA 02115;; ^c^Department of Dermatology, Harvard Medical School, Boston, MA 02115;; ^d^Department of Medical Oncology, Dana-Farber Cancer Institute, Boston, MA 02115;; ^e^Department of Medicine, Harvard Medical School, Boston, MA 02115;; ^f^Department of Chemical and Biomolecular Engineering, Vanderbilt University, Nashville, TN 37235;; ^g^Department of Molecular Physiology and Biophysics, Vanderbilt University, Nashville, TN 37235;; ^h^Department of Biochemistry and Molecular Biology, University of Chicago, Chicago, IL 60637;; ^i^Department of Biostatistics, Harvard T.H. Chan School of Public Health, Boston, MA 02115;; ^j^Department of Immunology, Faculty of Medicine, Universidad Complutense de Madrid, 28040 Madrid, Spain;; ^k^Department of Biomedical Engineering, Texas A&M University, College Station, TX 77843;; ^l^Department of Materials Science and Engineering, Texas A&M University, College Station, TX 77843;; ^m^Department of Physics and Astronomy, Texas A&M University, College Station, TX 77843;; ^n^School of Computational Sciences, Korea Institute for Advanced Study, 02455 Seoul, Korea

**Keywords:** T cell receptor, mechanosensor, optical tweezers, T cell activation, γδT cells

## Abstract

High-acuity αβT cell receptor (TCR) recognition of peptides bound to major histocompatibility complex molecules (pMHCs) requires mechanosensing, a process whereby piconewton (pN) bioforces exert physical load on αβTCR–pMHC bonds to dynamically alter their lifetimes and foster digital sensitivity cellular signaling. While mechanotransduction is operative for both αβTCRs and pre-TCRs within the αβT lineage, its role in γδT cells is unknown. Here, we show that the human DP10.7 γδTCR specific for the sulfoglycolipid sulfatide bound to CD1d only sustains a significant load and undergoes force-induced structural transitions when the binding interface-distal γδ constant domain (C) module is replaced with that of αβ. The chimeric γδ–αβTCR also signals more robustly than does the wild-type (WT) γδTCR, as revealed by RNA-sequencing (RNA-seq) analysis of TCR-transduced *Rag2*^−/−^ thymocytes, consistent with structural, single-molecule, and molecular dynamics studies reflective of γδTCRs as mediating recognition via a more canonical immunoglobulin-like receptor interaction. Absence of robust, force-related catch bonds, as well as γδTCR structural transitions, implies that γδT cells do not use mechanosensing for ligand recognition. This distinction is consonant with the fact that their innate-type ligands, including markers of cellular stress, are expressed at a high copy number relative to the sparse pMHC ligands of αβT cells arrayed on activating target cells. We posit that mechanosensing emerged over ∼200 million years of vertebrate evolution to fulfill indispensable adaptive immune recognition requirements for pMHC in the αβT cell lineage that are unnecessary for the γδT cell lineage mechanism of non-pMHC ligand detection.

## Significance

TCR mechanosensing is thought necessary for digital sensitivity of αβT cell response to scant pMHC antigens. We use bioinformatic analysis, molecular dynamics, single-molecule optical tweezers techniques, cellular activation, and RNA-seq analysis to explore this paradigm in the γδT cell lineage. We find that, in keeping with its role in recognizing abundant cell-surface ligands, the γδTCR lacks force-dependent hallmarks of mechanosensing in αβT cells.

Within jawed vertebrates (Gnathostoma), αβ and γδT cells utilize somatic genomic rearrangement to generate a receptor repertoire large enough to recognize the enormous diversity of viral or other pathogen-derived antigens and then mount a protective immune response (1–4). αβT cells are prominent in blood and lymph nodes while γδT cells are more abundant in barrier tissues including skin, intestinal and other epithelia, suggesting distinct, nonredundant roles for each T lineage subset (5, 6). Whereas the vast majority of αβT lymphocytes recognize sparse “foreign” peptides within a vast array of normal self-peptides processed and presented by classical major histocompatibility complex (MHC) molecules on the surface of pathogen-infected or otherwise damaged cells, this is almost without exception not the case for γδT cells (7–9). Instead, γδT cells recognize ligands, including self-derived and plentiful stress-induced nonpeptide ligands, diverse in structure and distinct from classical MHC molecules (6, 8, 10–17). Furthermore, γδT cells are generally in an activation state capable of mediating rapid (i.e., minutes- to hours-long time frames) innate-like cytolysis and cytokine release, in contrast to αβT cells, which exist in distinct naïve, effector, and memory states (6, 18). Naïve αβT cells become cytotoxic or mediate cytokine/chemokine-based inflammation only following differentiation that requires exposure over several days to antigen and costimulatory molecules on professional antigen-presenting cells (APCs).

Over the last decade, it has become increasingly clear that contrary to conventional receptor–ligand interactions exemplified by antigen–antibody binding, bioforces are essential for nonthermal equilibrium, mechanosensor-based αβT cell activation (19–26). αβT cell motility and the local cytoskeletal machinery place physical load on individual αβTCR–pMHC bonds, which tunes both the sensitivity and specificity of αβTCR recognition (19). In fact, chemical thresholds in the absence of external load require a 1,000- to 10,000-fold higher number of pMHC molecules than observed physiologically to trigger cellular αβT cell activation (20). In contrast, under force the ligand-mediated induction of the αβT cell biological response can be essentially digital. Mechanistically, physical load fosters stability and interfacial matching as well as a temporally correlated structural transition in the αβTCR heterodimer ectodomain that strengthens bond lifetime (i.e., a “catch bond”), energizes the αβTCR and induces αβTCR complex quaternary change, conformationally altering the transmembrane (TM) segments and lipids surrounding the TCR and thereby exposing the immunoreceptor tyrosine-based activation motifs (ITAMs) in the cytoplasmic tails of the associated signaling CD3 for phosphorylation (19, 27–29 and refs. therein). As the structural transition of the TCRαβ heterodimer is itself reversible in the context of relevant bioforces, a ligated TCR can be repetitively energized by the same pMHC in the absence of its release, thereby perpetuating the activation geometry of the αβTCR and surrounding membrane to optimize cognate antigen-dependent signaling performance, accounting for high acuity recognition (21, 30).

The role of bioforces in γδTCR recognition has not been addressed. Here, we examine the character of physical load in γδTCR signaling, comparing and contrasting with αβTCR signaling, using a combination of single-molecule (SM) optical tweezers-based technology, molecular dynamics (MD) simulation, transcriptomics, and functional analyses. For this purpose, we selected the DP10.7 γδTCR, since it is structurally well characterized and binds the MHCIb molecule, CD1d, in complex with a defined ligand, sulfatide. This TCR–ligand pair is similar in overall three-dimensional topology and size to complexes of peptides bound to MHCI K^b^ or D^b^ molecules used in related work in αβTCR systems (31). Our findings reveal that the DP10.7 γδTCR, unlike αβTCRs, does not bear the force-sensitive hallmarks of a functional mechanosensor. Instead, although DP10.7 binds ligand with submicromolar affinity under zero force, it readily dissociates from its ligand under force loading. By creating a chimeric Vγδ–Cαβ TCR in which Cαβ replaces Cγδ, we define a gain of function that supports mechanotransduction comparable to the level of a wild-type (WT) αβTCR. Moreover, RNA-sequencing (RNA-seq) analysis at the double negative (DN) thymocyte stages following retroviral transduction of TCRs into *Rag2*^−/−^ thymocyte progenitors reveals that such a chimeric TCR complex augments signaling compared to thymocytes expressing the wild-type γδTCR. Collectively, these findings inform that mechanotransduction associated with structural transitions and dynamic bond formation are linked to the constant domains in αβTCRs rather than a property of their variable domains per se. We reconcile our data with those of prior studies suggesting that γδTCR signaling is stronger than that of αβTCR during thymocyte development. Lastly, we discuss how structural differences between these two lineages of TCRs are well aligned with their distinct ligand specificities and attendant biology.

## Results

### The CβFG Loop Structurally Distinguishes αβ from γδTCRs.

**Fig. 1.**
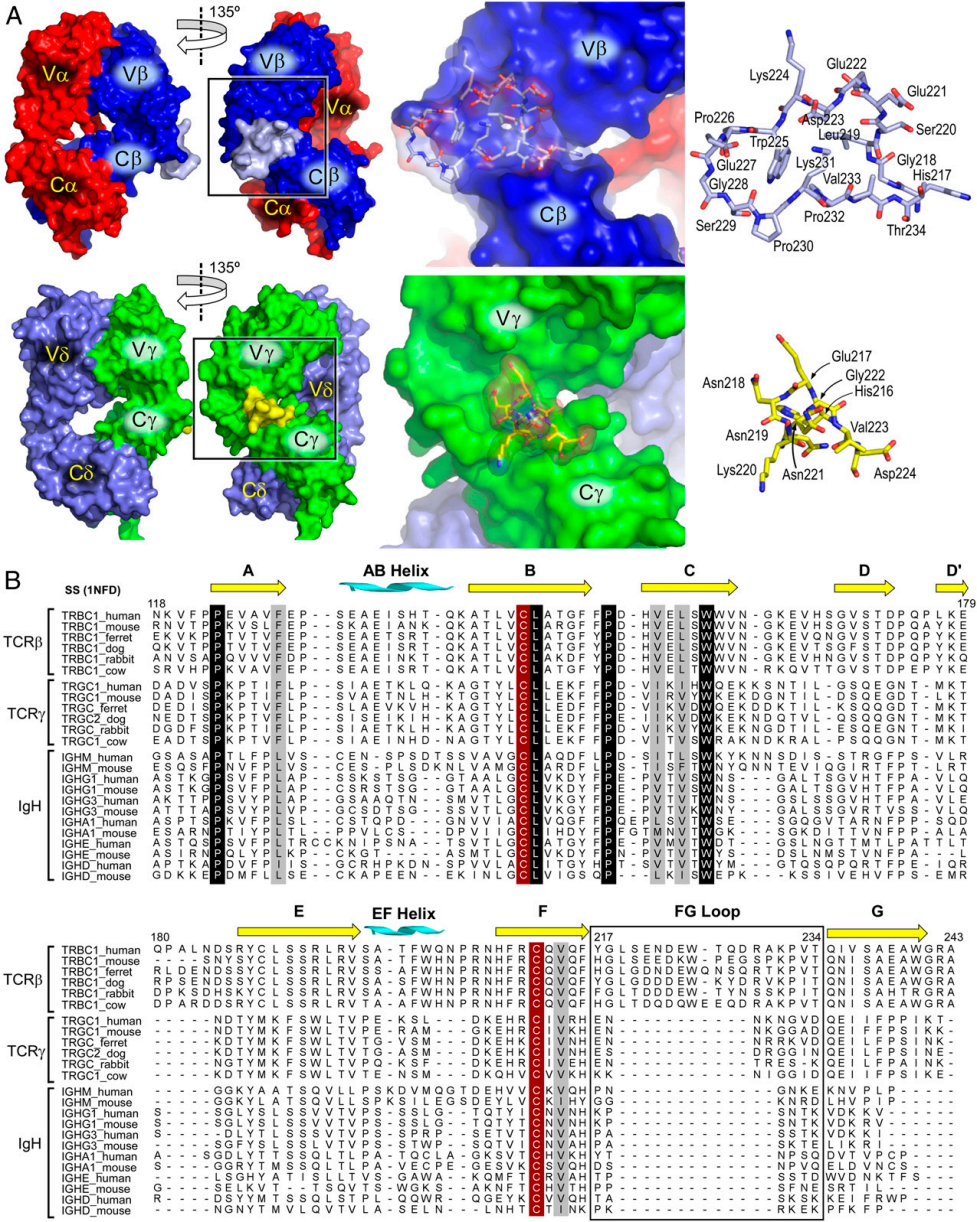
Structural features of γδ and αβTCRs. (*A*) Structural comparison of TCRαβ (*Top*) with TCRγδ (*Bottom*). The *Left* two panels in each row show surface representations with individual subunits and domains delineated. The FG loop is shown in light blue (TCRαβ) or yellow (TCRγδ) and highlighted in the boxed region. The *Right* two panels of each row present a zoomed view of the boxed region at approximately the same magnification for each TCR to illustrate the relative size and structure of the respective FG loops. (*B*) Multiple sequence alignment of mammalian TCR Cβ, Cγ domains, and equivalent Ig CH1 domains of selected isotypes. Secondary structure as assigned in the murine N15αβ X-ray structure 1NFD are indicated. The FG-loop region is boxed. Invariant cysteines are highlighted in red-brown and conserved residues denoted in black and gray.

We have previously characterized the mechanotransducing properties of the αβTCR at the SM and single-molecule–single-cell (SMSC) level and have noted signaling correlates with several structural features (19, 21, 27, 28, 30). Tied to preservation of the force-mediated catch bond, a requisite feature in pMHC ligand discrimination is the CβFG loop. We observe that the γδTCR, while sharing many features with the αβTCR, including CD3 components and general ectodomain organization, lacks the large FG loop in its homologous Cγ domain as compared to the CβFG loop within the αβTCR (Fig. 1*A*). The CβFG loop but not that of Cγ appears to buttress the region joining the V and C domains (Fig. 1*A*). Multiple sequence alignment of mammalian C domains, comparing Cβ and Cγ with C regions of human or murine immunoglobulin heavy (IgH) subtypes clearly shows that the extensive FG loop (containing a 12- to 13-amino-acid insertion relative to the heavy chain constant region domain 1 [CH1]) is a conserved feature only within the αβTCR (Fig. 1*B*). This contrasts with otherwise high structural conservation throughout the domains. Lack of the distinct FG loop feature within Cγ could plausibly indicate either that equivalent mechanotransduction is not a property of the γδTCR, or alternatively that Cδ may provide the avenue of mechanosensing, particularly given the predominance of the Vδ in several characterized γδTCR–ligand interactions (10, 31, 32).

### CβFG Loop Stabilizes the V–C Interface in αβ and Chimeric γδ–αβTCRs Relative to γδTCR.

**Fig. 2.**
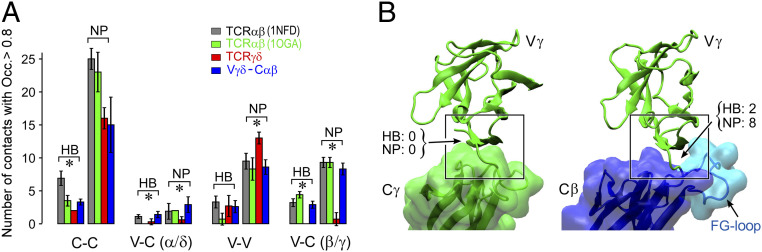
Comparison of interdomain contacts within TCRγδ, TCRαβ, and TCRγδ–αβ chimera. A 100- to 300-ns interval during MD simulation for each system was used for analysis (*Materials and Methods*). (*A*) Average number of contacts with occupancy greater than 80% (bar: SD of measurements in 10 overlapping time windows of size 36.4 ns). HB, hydrogen bond; NP, nonpolar contact. Locations of these contacts within each structure are shown in *SI Appendix*, Fig. S1. For the Cγ–Cδ interface, two hydrogen bonds were counted in all 10 windows, hence it has no error bar (red in the first HB group). Asterisk shows average number of contacts between TCRγδ and the TCRγδ–αβ chimera differing with significance level smaller than 10^−5^. (*B*) Comparison of the Vγ–Cγ interface and the Vγ–Cβ interface. Constant domains have surface representations overlaid in semitransparent colors, as approximate markers for their boundaries. Number of contacts of occupancy greater than 80% are marked (cf., *SI Appendix*, Fig. S1). Among the 10 Vγ–Cβ interface bonds, the CβFG loop contributes one H bond and three nonpolar contacts. Boxes highlight the difference in conformations between the two systems, where the valley created by the FG loop in Cβ helps with stabilizing Vγ.

As we (19, 33) and others (34) have noted, the CβFG loop provides significant structural contact between Vβ and Cβ that is absent in the antibody V–C interface (∼350 Å^2^ versus ∼150 Å^2^ buried surface areas, respectively). Our previous MD simulation on TCRαβ showed that the CβFG loop influences the motion of the variable domains relative to the constant domains, as well as imposes an orientational restraint of the former while facing pMHC, which enables a load- and ligand-dependent control of the bond lifetime (29). Comparative MD simulations of the TCR structures used in the present study show that the Vγ–Cγ interface has significantly fewer high-occupancy contacts as compared to Vβ–Cβ [Fig. 2*A*, V–C(β/γ); also see *SI Appendix*, Fig. S1], and similar to the α-chain of TCRαβ, the δ-chain has few V–C contacts [Fig. 2*A*, V–C(α/δ) group]. Thus, the TCRγδ constant domains are unlikely to have any strong influence on the conformational motion of the variable domains. Another property important for mechanosensing in TCRαβ is the compliance of the Vα–Vβ interface, leading the interface with pMHC to be responsive to and controlled by an applied load. In our previous simulation, TCRαβ lacking the CβFG loop had an increased number of Vα–Vβ contacts (29). The higher number of nonpolar Vγ–Vδ contacts (Fig. 2*A*, V–V group) also suggests a reduced compliance, which further makes TCRγδ unsuitable for mechanosensing. In contrast, when Cαβ was substituted for Cγδ the number of Vγ–Cβ contacts increased, comparable to that of the full TCRαβ [blue in Fig. 2*A*, V–C(β/γ), *SI Appendix*, Fig. S1], perhaps explaining why chimeric Vγδ–Cαβ ectodomain constructs have been successfully utilized in structural studies of TCRγδ (31). The CβFG loop structurally supports the Vγ domain as well, forming nearly half of its high-occupancy contacts (Fig. 2*B*). Our simulations thus suggest that not only will the γδTCR lack the requisite allosteric connections for mechanotransduction, but also that these connections should form in chimeric Vγδ–Cαβ constructs mainly via nonpolar contacts (*SI Appendix*, Fig. S1). By this analysis it would also appear that the Vδ–Cδ interface is unlikely to compensate for the relative paucity of contacts in the Vγ–Cγ interface and shift the mechanosensing potentiation to the δ-subunit.

### The γδTCR Lacks Strong Mechanotransduction Elements, which Are Rescued by Replacement of γδ C Domains with Those of αβ.

**Fig. 3.**
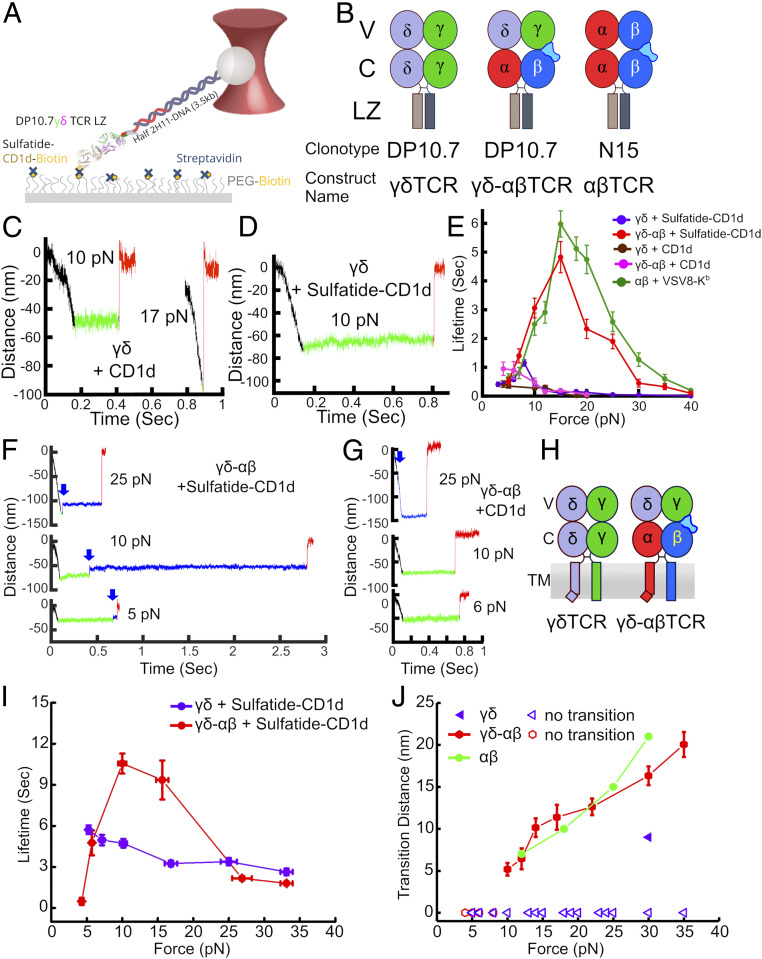
SM and SMSC measurement of TCRγδ DP10.7–CD1d interaction. (*A*) LZ-coupled TCR is bound to acid-base LZ-specific half-mAb 2H11 coupled to a DNA linker attached to a polystyrene bead held in an optical trap. Biotinylated CD1d is bound to streptavidin, which is itself bound to PEG–biotin that is attached to the movable piezo stage. (*B*) Constructs used in SM experiments. (*C*) Representative SM traces for DP10.7 interaction with CD1d lacking exogenous ligand (CD1d) at 10 or 17 pN. Force load is applied in the black section of the trace, binding dwell is the green section and bond dissociation is the red section. (*D*) SM trace of DP10.7 interaction with CD1d bound to sulfatide (sulfatide–CD1d) with 10 pN pulling force. (*E*) Force vs. lifetime plot for the DP10.7 TCRγδ (purple curve, *n* = 191) or DP10.7γδ–αβ chimera (red curve, *n* = 126) interaction with sulfatide–CD1d, TCRγδ (brown curve, *n* = 101) or γδ–αβ (pink curve, *n* = 92) with CD1d, or N15αβ interaction with its cognate ligand VSV8/H-2K^b^ (30) (green curve, *n* = 192). Error bars indicate SEM. (*F*) SM traces at indicated forces for DP10.7γδ–αβ chimera interaction with CD1d–sulfatide. Initial binding dwell is shown in green and posttransition dwell in blue. Transition points are indicated in each trajectory with blue arrows. Black and red sections are as in *C*. (*G*) SM traces at indicated forces for DP10.7γδ–αβ chimera interaction with CD1d with color coding as in *F*. A transition was identified in the 25-pN trace (blue arrow). (*H*) Constructs used in SMSC optical trap assay are indicated. Note that the TCRδ TM is depicted with a bend analogous to that of TCRα (28), although there is currently no data to confirm either a bipartite or single helix structure. (*I*) SMSC optical trap assay force vs. lifetime plot for the DP10.7 TCRγδ interaction (purple curve, *n* = 45) or DP10.7γδ–αβ chimera (red curve, *n* = 44) interaction with sulfatide–CD1d. Error bars indicate SEM. (*J*) Transition distances for DP10.7γδ–αβ chimera (red curve, 23 of 44) interaction with sulfatide–CD1d from traces acquired by SMSC as compared to N15αβ (19) (green curve, *n* = 15). Error bars indicate SEM. For reference only a single, shorter transition was found in 45 traces for DP10.7 TCRγδ interaction with sulfatide–CD1d (purple triangles, see key).

To experimentally determine whether or not the TCRγδ has the potential for mechanosensing by direct biophysical analyses, we utilized the DP10.7 γδTCR (DP10.7γδ) in SM experiments to test the hypothesis that the γδTCR is a mechanosensor (Fig. 3*A*). Sulfatide–CD1d is the preferred ligand for DP10.7 with only weak interactions with CD1d in the absence of sulfatide lipid (31). To probe the DP10.7 TCRγδ–ligand interaction, CD1d ± sulfatide was bound to a functionalized surface in a tethered bead configuration (19) (Fig. 3*A*). To this end, DP10.7γδ was cloned and produced as a leucine zipper-paired heterodimer (LZ) with N15αβ–VSV8/K^b^ used as comparison (Fig. 3*B*) (19, 31). CD1d or VSV8/K^b^ ligands were bound through streptavidin to a PEG-pacified surface containing sparse PEG–biotin. The TCRs fused to LZ at the C terminus were tethered via a long DNA molecule such that force could be applied to the optically trapped bead (*Materials and Methods* and ref. 19). Lifetime measurements were performed by translating each sample relative to the fixed trap using the piezo stage, then holding at a fixed position/force until bond rupture. Bond rupture is identified as an abrupt snap back of the bead to the trap center (distance = 0 nm) while conformational extensions are observed as smaller displacements of the bead toward the trap center.

Testing the WT DP10.7 against CD1d alone in the absence of sulfatide we found lifetimes of less than 0.5 s down to 0.3 s at 10 pN (Fig. 3*C* and *SI Appendix*, Fig. S2 *A* and *B*) while sulfatide-complexed CD1d resulted in similar bond lifetimes with the exception of a narrow force window in which lifetimes around 1 s were measured (Fig. 3*D* and *SI Appendix*, Fig. S2*C*). In the absence of sulfatide loading, bond formation was of lower frequency, with 1 to 2 tethers per field of view found in comparison to ∼25 when presented with sulfatide–CD1d. Whereas the CD1d without exogenous ligand resulted in a slip-bond profile in force-bond lifetime plots of the collected binding data, a narrow enhancement of bond lifetime was measured for sulfatide–CD1d with forces under 10 pN, suggesting force may minimally organize the interface (29). There was no continued enhancement above 10 pN; instead, the system transitioned to a slip bond. This force threshold corresponds to loads generated by cells even under static culture conditions, and so this does not appear to be a physiologically relevant catch bond as observed in other systems (19, 26, 35–37) (Fig. 3*E*). In contrast, when the chimeric γδ–αβTCR is tested versus sulfatide–CD1d, longer bond lifetimes were measured (Fig. 3*F* and *SI Appendix*, Fig. S2*D*) with organization/bond strengthening continuing on to form a typical catch bond of 5-s lifetime at 15-pN force, comparable to N15αβ interacting with its cognate ligand VSV8-K^b^ (Fig. 3*E*). Incorporating the Cαβ domain in lieu of Cγδ thus leads to force-responsive properties in the context of the same Vγδ domain module and ligand. Of note, γδ–αβTCR does not form a catch bond with CD1d lacking sulfatide (Fig. 3 *E* and *G* and *SI Appendix*, Fig. S2*E*) in keeping with previous observations of ligand gating specificity by TCRαβ and, more specifically, requisite allosteric control within Cβ, another hallmark of mechanosensing (19, 26, 29, 30). Indeed, the SM sensitivity index (the ratio of a TCR–ligand bond lifetime for specific to nonspecific MHC-bound interaction) (19) shows discrimination of the γδ–αβTCR to be much greater than that of the γδTCR, and more similar to the αβTCR (*SI Appendix*, Fig. S3). The appearance of sustained bond lifetime under force was consistently accompanied by a structural transition for the γδ–αβTCR (Fig. 3*F*), analogous to that seen previously for the αβTCR and pre-TCR in response to specific ligands (19, 30) but essentially unobserved in traces for the γδTCR (Fig. 3 *C* and *D* and *SI Appendix*, Fig. S2*C*).

### Catch Bond and Structural Transition Rescue by αβ C Domains on the T Cell Surface.

To confirm that the biomechanical observations above translate to the fully assembled and membrane-embedded γδTCRs on T cell surfaces, TCR γδ or γδ–αβ heterodimers (Fig. 3*H*) were retrovirally transduced and resultant TCRs were interrogated with bead-bound sulfatide–CD1d in the SMSC format as previously described (19). To this end, BW5147 T cells lacking endogenous TCRs were transduced using a 2A peptide-containing construct for simultaneous expression of paired TCR subunits (38). Murine C domains and TM regions were substituted for the human counterparts to attain optimal surface expression in this mouse-derived thymocyte lymphoma line as previously described (39, 40). As shown in Fig. 3*I*, the γδTCR exhibits only slip bond, whereas in contrast, the chimeric γδ–αβTCR manifests a significant catch bond up to 15 pN and slip bond thereafter, in parallel to the SM behavior, demonstrating the same force sensitivity. The significant bond lifetime over a range of forces for both TCRs is likely due to the presence of CD3 molecules in the TCR complex (ref. 28 and refs. therein) as well as an increase in compliance and stress relaxation of the cell membranes and connecting linkages compared to the biotinylated surfaces in the SM assay (Fig. 3*I*). The SM is more similar to a force clamp, while the SMSC behaves more like a stress relaxation test. The cell mechanical linkage pathway may also participate in modulating bond lifetime, even actively through actin–myosin-based coupling and feedback (20). We note that SM and SMSC assays are executed differently, yielding a slower effective “instrument response time” for SMSC. In the absence of sulfatide we observed lower frequency of bond formation, with fewer than 10% of beads forming effective tethers for either γδ (1 in 25) or γδ–αβ (2 in 25), as compared to ∼50% when presented with CD1d–sulfatide. Moreover, the structural transition previously observed in αβTCRs and pre-TCRs (19, 30) is present here in over 50% (23/44) of the individual traces and is similar in length to that of the N15αβ TCR (19) (Fig. 3*J*). All transitions occurred at 10 pN or higher force for the chimeric γδ–αβTCR, consistent with behavior of the N15αβ TCR (19). In contrast, only 1 in 45 traces had evidence of a transition for the γδTCR. These data strongly suggest that the γδTCR lacks specific adaptations for mechanosensing, a result consistent with absent force-dependent ligand signaling threshold sensitivity enhancement (*SI Appendix*, Fig. S4).

### Enhanced Thymic Signaling through TCR Is Imparted by αβ C Domains.

**Fig. 4.**
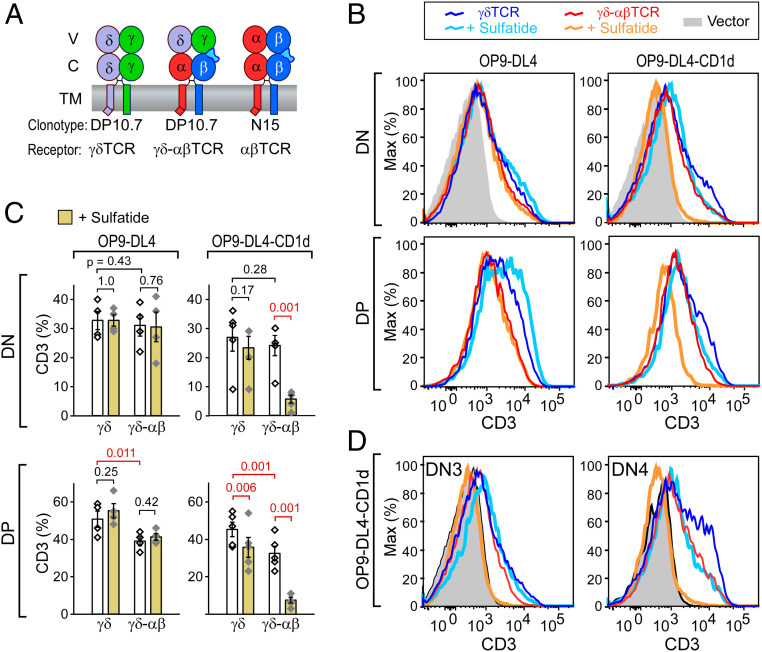
Thymocyte response to ligand in stromal cultures. (*A*) Constructs used in in vitro thymic stromal culture. (*B*) Surface CD3 FACS fluorescence-activated cell sorting (FACS) analysis of DP10.7γδ (γδTCR) or DP10.7γδ–αβ (γδ–αβTCR)-transduced thymocytes cultured for 8 d in the absence or presence of sulfatide in coculture with parental OP9–DL4 stromal cells or OP9–DL4 cells stably transfected with human CD1d (OP9–DL4–CD1d). All cells were gated with FSC-A and SSC-A to isolate thymocytes then GFP^+^CD45^+^ to select transfected thymocytes. CD4^+^CD8^+^ = DP; CD4^−^CD8^−^ = DN (*SI Appendix*, Fig. S6*A*). Note: DP thymocytes fail to develop with vector transduction only. (*C*) Statistical analysis of five independent experiments as represented in *B*. Significance (*P* value) was determined by linear regression analysis. White bars are from cultures treated with dimethyl sulfoxide vehicle only, yellow are sulfatide treated. (*D*) Surface CD3 FACS analysis of DN3 and DN4 subsets in response to sulfatide ligand in OP9–DL4–CD1d stromal cell culture. Histogram colors are as in *B*.

To assess the biological consequences of TCR mechanosensing function, we exploited an in vitro stromal cell-lymphoid progenitor coculture experimental system. Fetal liver–derived thymic progenitors isolated from *Rag2*^−/−^ B6 mice were transduced with either full-length γδTCR, γδ–αβTCR, or αβTCR heterodimers as detailed in Fig. 4*A*. Stromal cells used in this assay were OP9–DL4 applied previously for both γδT cell and αβT cell development (35, 41). In order to test the effect of ligand binding, an OP9–DL4 cell line, which expresses single-chain human CD1d/β2m at levels comparable to those of murine CD1d on these same cells, was developed (*SI Appendix*, Fig. S5 *A* and *B*). DP10.7 tetramer binding analysis (31) confirmed robust binding to this OP9–DL4–CD1d cell line when exogenous sulfatide was added (*SI Appendix*, Fig. S5*C*). Some tetramer binding was detected for the OP9–DL4–CD1d cell line in the absence of sulfatide addition. WT OP9–DL4 + sulfatide showed a slight increase over untreated WT OP9–DL4, while the OP9–DL4–MHC knockout (KO) cell line, which lacks class I MHC expression (30) including CD1d, showed no detectable effect of sulfatide treatment (*SI Appendix*, Fig. S5*C*).

Following an 8-d coculture of γδ or γδ–αβ TCR-transduced DN3 *Rag2*^−/−^ thymocytes with either WT OP9–DL4 or OP9–DL4–CD1d, we observed transition of thymocytes from the CD4^−^CD8^−^CD25^+^CD44^−^ (DN3) population through CD4^−^CD8^−^CD25^−^CD44^−^ (DN4) with a subset also progressing beyond DN4 to CD4^+^CD8^+^ (DP) (*SI Appendix*, Fig. S6*A*). When sulfatide was added to each culture, a differential loss in surface CD3 staining was measured in association with human CD1d-expressing stromal cells (Fig. 4*B*). This effect was significantly more pronounced for γδ–αβTCR transduced thymocytes versus those expressing WT γδTCR and is present both in DN and DP thymocytes (Fig. 4 *B* and *C*). Surface CD3 loss is most likely due to an enhanced responsiveness of these thymocytes imparted by the αβTCR C regions leading to down-modulation of the TCR complex with activation (42). The down-modulation occurs in both DN3 and DN4 subsets for γδ–αβTCR, while the effect is not observed for γδTCR in DN3 (Fig. 4*D*). Of note, post-DN4 cell numbers following culture were significantly reduced for the γδ–αβTCR cultures on stroma with sulfatide present (*SI Appendix*, Fig. S6 *B* and *C*). This was not the case for γδTCR thymocytes. In addition, the OP9–DL4 stroma expressing endogenous murine CD1d-only also showed this reduction, consistent with the ability of 10.7 TCR tetramers to bind weakly but nevertheless clearly to OP9–DL4 (*SI Appendix*, Fig. S5*C*). Loss of DP thymocytes in γδ–αβTCR- but not γδTCR-transfected thymocytes on both OP9–DL4 stroma implies negative selection linked to the CαCβ module (*SI Appendix*, Fig. S6*C*).

The strong down-regulation of CD3 on γδ–αβ TCR transduced thymocytes following sulfatide exposure implied that active TCR signaling was occurring for γδ–αβTCR thymocytes. Given that higher TCRγδ–ligand signaling strength was reported to induce IFNγ as opposed to IL-17 T cell differentiation (43–45), we assayed cytokine production within hours of calcium ionophore plus phorbol myristate acetate (PMA) stimulation following 8 d of γδTCR and γδ–αβTCR thymocyte–stromal cultures with or without exogenous sulfatide addition (*SI Appendix*, Fig. S7). While no enhanced IFNγ production was observed when comparing γδ–αβTCR to γδTCR, an absence of cellular elements in epithelial cultures present in thymus and required to recapitulate the cytokine phenotype could not be excluded. As a consequence, we selected a more global and unbiased approach to interrogate signaling differences.

### Chimeric γδ–αβTCR Signaling Generates Stronger Transcriptome Changes than those Through WT γδTCR.

**Fig. 5.**
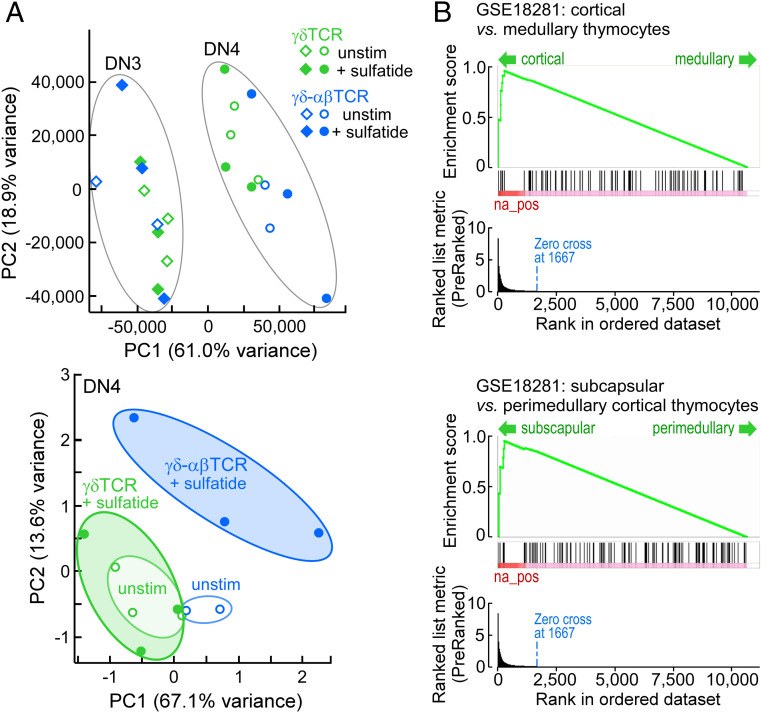
Transcriptome analysis of DN3 and DN4 transduced thymocytes after 8 d of coculture with OP9–DL4–CD1d stromal cells in the presence or absence of sulfatide. (*A*) Global PCA of all populations delineates DN3 and DN4 populations (*Upper*). Restricted PCA of DN4 cells separates the cell states independent of the DN3-to-DN4 drivers (*Lower*). Ellipses in both panels provide a visual measure of overlap or separation of the indicated populations. Data represent three independent experiments for each condition except for γδ–αβTCR unstimulated (*n* = 2). (*B*) Gene set enrichment analysis (MSigDB C7 immune signatures) identifies the DN4 γδ–αβTCR sulfatide-stimulated transcriptome signature as being consistent with that of cortical thymocytes (*Upper*) with a subcapsular location (*Lower*).

To this end, we analyzed transcriptome signatures of DN3 and DN4 thymocytes from lymphoid progenitors previously transduced with γδTCR or γδ–αβTCR and then cultured on OP9–DL4–CD1d with or without sulfatide addition for 8 d. Three independent experiments analyzing DN3 and DN4 cells ± sulfatide for the γδTCR- and γδ–αβTCR-transduced thymocytes yielded 24 cDNA libraries that underwent next generation sequencing (Dataset S1 and *SI Appendix*, Tables S1–S7). Global principal component analysis (PCA) separated cleanly between the DN3 and DN4 populations (Fig. 5 *A*, *Upper*) where the drivers of this transition (e.g., *Samhd1*, *Lgals3*, *St3gal6*, *Gpr15*, and *S100a4*; Dataset S1 and *SI Appendix*, Table S4) dominated the PCA, masking underlying transitions induced by the response of the wild-type and chimeric TCR to CD1d–sulfatide. Nevertheless, at the DN3 stage, only 20 genes were significantly fold-change regulated in γδTCR thymocytes following sulfatide stimulation (Dataset S1). In contrast, in DN3 γδ–αβTCR-expressing thymocytes responding to sulfatide, similar changes were observed not only for the 20 altered in the γδTCR cells but for >4,000 other genes (Dataset S1 and *SI Appendix*, Table S5). PCA alone, however, could not separate the DN3 γδTCR and γδ–αβTCR sulfatide-stimulated populations as the gene expression patterns were almost identical (Fig. 5 *A*, *Upper* and Dataset S1); the differences resided in the extent of the fold change. Additionally, even in the absence of exogenous sulfatides, the CαCβ domains in the γδ–αβTCR-expressing thymocytes influenced the signaling background. For example, using *Cd69* up-regulation as a proxy for TCR stimulation (46), the γδTCR background for *Cd69* at the DN3 stage was 153.6 ± 25.4 expression reads while, in γδ–αβTCR, levels were 218.3 ± 26.6 with both rising to ∼270 on sulfatide stimulation with a similar representation at the DN4 stage (Dataset S1 and *SI Appendix*, Table S1).

Analysis at the DN4 stage, however, removing the influence of the DN3 to DN4 drivers, permitted discrimination of significant differences in expression programs for γδ–αβTCR and γδTCR (Fig. 5 *A*, *Lower*). For the unstimulated condition, PCA revealed a slight shift on the PC1 axis for γδ–αβTCR, likely representing the background elevation of discrete gene transcripts, as discussed further below. Upon addition of sulfatide, the γδ–αβTCR DN4 program shifts strongly on the PC2 axis as well as on the PC1 axis. In contrast, the centroids for the γδTCR-unstimulated and γδTCR-stimulated populations appear similar. Immune gene expression analysis (47), a system trained on subsets of mature single-positive CD4 and CD8 cells both resting and responding to a variety of stimuli, as well as on phenotypically defined and thymic region-localized thymocyte populations, identifies the DN4 γδ–αβTCR sulfatide-stimulated population as highly and significantly similar to subcapsular cortical thymocytes (Fig. 5*B*).

**Fig. 6.**
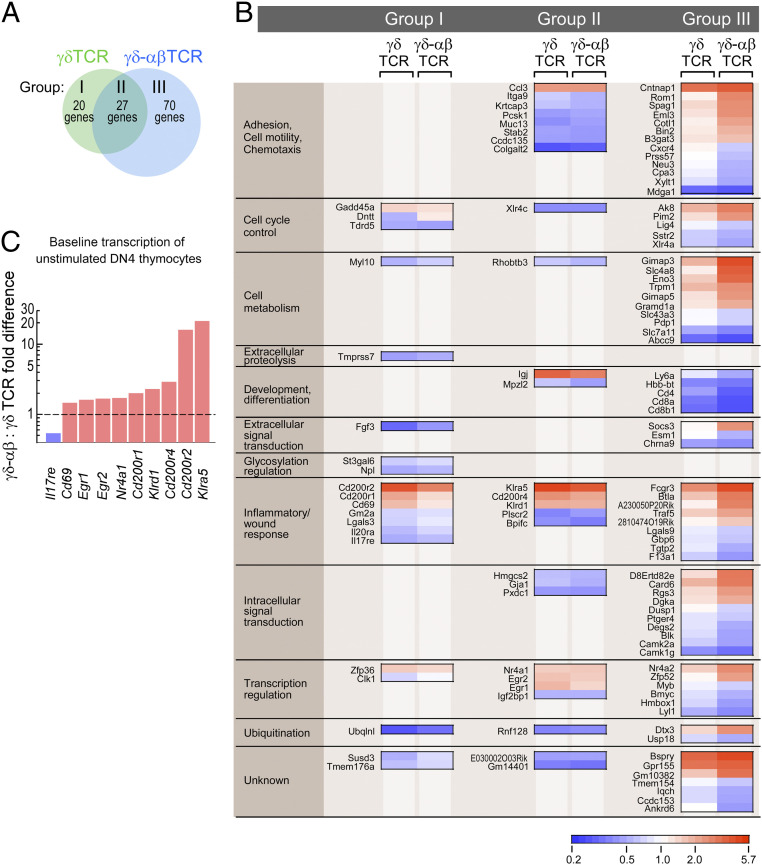
Gene signatures for DP10.7γδ (γδTCR) or DP10.7γδ–αβ (γδ–αβTCR) control and sulfatide-stimulated states. (*A*) For DN4 thymocytes bearing γδ or γδ–αβ TCRs and developing on OP9–DL4–CD1d stromal cells in the absence or presence of sulfatide, RNA was isolated and gene expression profiles were determined by RNA-seq. For each TCR, gene expression profiles delineating the stimulated from the control state were determined using a threshold for *p.adj* ≤ 0.1. Gene signatures were defined as present only in γδTCR-bearing cells (group I), only in γδ–αβTCR-bearing cells (group III), or shared in both conditions (group II). (*B*) Heat map profiles, ordered into functional groupings (*Left* column) for the genes in developing γδ–αβTCR DN4 thymocytes identified as being significantly regulated in the presence of sulfatide. Expression profiles for the same genes developing in γδTCR-bearing thymocytes are also depicted. The scale indicates fold reduction (blue) or fold increase (red). White indicates no fold difference between control and stimulated state. For group I, the fold differences after stimulation did not differ significantly between γδTCR and γδ–αβTCR (*P* = 0.069) but for all γδTCR transcripts, *p.adj* ≤ 0.1 and for all γδ–αβTCR, *p.adj* > 0.1. For group II, *p.adj* ≤ 0.1 for all indicated genes with no significant difference in fold change. For group III, only the γδ–αβTCR transcripts have a *p.adj* ≤ 0.1 with an overall significant fold change over γδTCR (*P* < 0.0001). (*C*) For select genes in group I and group II, fold differences between γδ–αβTCR- and γδTCR-bearing thymocytes in the unstimulated control condition are presented. The dashed line delineates identity between γδTCR- and γδ–αβTCR-unstimulated expression levels. For all fold differences depicted (pink for γδ–αβTCR > γδTCR, blue for γδ–αβTCR < γδTCR), *P* < 0.05.

Comparison of the gene expression profiles for γδTCR and γδ–αβTCR thymocytes responding to sulfatide at the DN4 stage reveals 117 significantly regulated gene transcripts for which 20 appear to be preferentially regulated in the γδTCR condition (group I), 27 are shared (group II), and 70 are preferentially regulated in the γδ–αβTCR condition (group III) (Fig. 6*A* and Dataset S1). Analysis of the γδ–αβTCR group III profile, with significantly regulated transcripts grouped according to functional activity, shows that many of the gene transcript levels are directionally regulated similarly in γδTCR-expressing thymocytes but that the degree of regulation is much greater in γδ–αβTCR-expressing thymocytes (Fig. 6*B*). Several of the genes represented in group I (specifically *Cd69*, *Egr1*, *Egr2*, *Nr4a1* [*Nur77*], *Cd200* variants and *Klrd1* [*Cd94*], and *Klra5* [*Ly49e*]) are key markers for TCR stimulation (43, 46, 48–52). Given that TCR signaling, measured as reduced cell surface expression of CD3, was greater in the γδ–αβTCR condition than in the γδTCR condition, the inclusion of such TCR signaling-associated transcripts in group I was unexpected. As depicted in Fig. 6*C*, however, genes expected to be down-regulated or up-regulated upon TCR signaling had already moved in these respective directions in the γδ–αβTCR cells in comparison with γδTCR cells prior to sulfatide stimulation. Since the maximal stimulation changes for this signaling-associated gene group were similar for both cell types, the apparent fold change upon sulfatide stimulation is greater for the γδTCR cells than for the γδ–αβTCR cells. In the absence of exogenous sulfatides, the background binding of DP10.7 TCRγδ tetramer is greater to OP9–DL4–CD1d than parental OP9–DL4 (*SI Appendix*, Fig. S5). Hence, endogenous sulfatide presentation may be sufficient to stimulate those genes noted in Fig. 6*C* through the chimeric γδ–αβTCR but not the γδTCR before signaling induction by exogenously added sulfatides. Overall, the transcriptomics results support the notion of enhanced signaling sensitivity and function of the γδ–αβTCR.

## Discussion

Our results demonstrate that γδT lineage cells exhibit differential signaling sensitivity to αβ T lineage cells by virtue of their respective C modules. The relevance for γδT cell biology generally, and analog signaling in particular, is highlighted below. Pointedly, γδT cells cannot exploit TCR mechanosensing as used by αβT cells to harness physical load and extend TCR–ligand bond lifetime under nonequilibrium conditions. Load in vivo results from a range of bioforces that lymphocytes experience during both external (cell movement) or internal (cytoskeletal rearrangement) processes (reviewed in ref. 21). This differential behavior is striking, given shared use of CD3 dimeric signaling components by both TCRs (ref. 27 and refs. therein), although some distinctions in CD3 heterodimers, FcεRIγ and CD3ζ composition, may further tune signaling (53–56). Replacement of CγCδ in WTDP10.7 γδTCR with CαCβ in the γδ–αβTCR construct confers αβTCR mechanosensing properties to the chimeric receptor whose VγVδ module (i.e., ligand interaction surface) remains the same. MD simulations show that the Vβ–Cβ interface is stabilized by the CβFG loop unique to mammalian pre-TCRs and αβTCRs (29) and thus likely implicates the β-subunit in this gain of function. In this regard, our earlier studies using optical tweezers on isolated TCR αβ heterodimers as well as αβTCR complexes on T lymphocytes documented how the VαVβ module was allosterically controlled by the CβFG loop (19, 30) to dictate TCR–pMHC bond lifetime as well as peptide discrimination. Concordantly, deletion of the CβFG loop created αβTCRs, whose recognition and signaling function were attenuated in vitro and in vivo (57, 58). αβTCR mechanosensing affords virtually digital responsiveness in signaling; only one or a handful of TCR–pMHC interactions is required for cellular activation, while chemical thresholds in the absence of load require pMHC numbers higher by orders of magnitude to trigger a cellular response (20). The pre-TCR, consisting of a pTα–β heterodimer, also manifests mechanosensing function that is CβFG loop dependent (30, 35).

Within Gnathostomata there was coevolution of the elongated CβFG loop and molecular speciation of CD3γ and CD3δ genes from a single CD3 precursor (58, 59). Thus, mammals, but likely not birds, amphibians, reptiles, or bony fish, are capable of mechanosensing in their respective αβTCR lineages. Given that the evolutionary distance between human and mouse from a common mammalian ancestor is 75 million years and that between human and birds from a common vertebrate ancestor is 300 million years, roughly 200 million years of vertebrate evolution was required for mechanosensing to emerge as the solution within the αβT lineage system to high acuity adaptive immune recognition.

Catch bonds are observed over a wide range of receptor–ligand systems (60). In these systems, the receptor is assumed to take two alternate conformations, one with a low affinity and the other with a high affinity to the ligand. Without load, the low-affinity state is prevalent. An applied load causes a conformational change to the high-affinity state, thereby initiating the catch-bond pathway (61). An essential aspect in this mechanism is allostery, where load-induced conformational change of the receptor alters the ligand-binding domain. While alternate conformations responsible for the two states have been observed in other systems (60), the structural origin for the catch bond behavior in TCRαβ has remained enigmatic, as no clear conformational states have been observed in X-ray structures of TCRαβ that might affect its interaction with the pMHC molecule. Two recent studies propose a catch-bond mechanism based on behaviors of a few hydrogen bonds between TCRαβ and pMHC that formed when the complex was rapidly pulled apart in MD simulation (36, 62). Aside from the use of very large forces within short simulation times where conformational relaxation cannot occur, analyses based only on a handful of transiently formed contacts cannot address the question of allostery, which involves conformational motion of the whole protein.

More recently, our own MD simulation study has illuminated a possible mechanism of catch-bond formation within the Vαβ–pMHC interface that is potentiated by V–C interactions, particularly those at the Vβ–Cβ interface, including the CβFG loop (29). An essential aspect of this mechanism is that the capacity to activate the catch bond is endowed by the conformational properties of the entire TCRαβ chassis rather than only by residues that immediately contact pMHC. More specifically, the four-domain organization leads to relative motion between Vα and Vβ, which can be stabilized by the applied load and in the presence of the cognate antigenic peptide. The CβFG loop is a structural element that is crucial for the allosteric control. By forming additional contacts with Vβ, the CβFG loop not only influences the Vα–Vβ motion, but it also supports its orientation amenable to form an interface with pMHC in loaded conditions.

Based on the above, the MD simulations in the present study indicate that the TCRγδ chassis is not well suited for mechanosensing. In particular, the Vγδ and Cγδ domains do not include sufficient contacts to establish allostery responding to load (Fig. 2 and *SI Appendix*, Fig. S1). Further, the greater nonpolar contacts between Vγ and Vδ suggest a lower compliance. In contrast, the Vγδ–Cαβ chimera has the number of contacts between Vγ and Cβ, as well as between Vγ and Vδ comparable to those for the corresponding interfaces of TCRαβ (Fig. 2), which is fully consistent with our experimental results, demonstrating the chimera responding to load similar to TCRαβ. The reduction in the number of Vγ –Vδ contacts is due to the orientational constraint imposed by the CβFG loop, as observed between the wild-type TCRαβ and a mutant lacking the CβFG loop (29). Since a majority of the contacts between Vγ and Cβ are nonpolar, a steric constraint imposed by the CβFG loop is likely more important than forming specific contacts (Fig. 2*B*). To further elucidate the steric nature of the contact, we built a model of Vαβ–Cγδ chimera and performed MD simulation (*SI Appendix*, Fig. S1*E*). There were little contacts at the Vα–Cδ interface, and a small number of contacts formed at the Vβ–Cγ interface. Since extensive contacts form at the Vβ–Cβ interface of TCRαβ, some of the residues in Vβ are amenable to form nonpolar contacts with Cγ in the chimera. However, the contacts are not extensive and we do not expect the Vαβ–Cγδ chimera to exhibit a catch bond as strongly as the Vγδ–Cαβ chimera. The increase in the Vα–Vβ contacts in this case is also consistent with the behavior of the FG-loop deletion mutant where the Vα–Vβ motion is suppressed (29). While additional insight will be gained from future simulations of TCRγδ complexed with sulfatide–CD1d, the present simulations elucidate conformational properties of TCRs that facilitate understanding of the current experiments.

The conserved γδT cell lineage chassis implies that mechanosensing is not a feature of γδTCRs, but detailed assessment of other TCRs using biophysical methods performed here is warranted. That said, TCR ligation-based exposure of the CD3ε proline-rich cytoplasmic region in αβTCRs but not γδTCRs mapping to their respective constant regions reported previously (63) is consistent with the generality of differential mechanotransduction revealed here.

Anticipating that mechanosensing would augment TCR signaling upon sulfatide exposure, we observed that the chimeric γδ–αβTCR-transfected thymocytes showed greater activation than the WT γδTCR-transfected thymocytes in CD1d–OP9 DL4 epithelial cultures at DN stages and beyond. This was initially revealed as a reduction in cell surface CD3 expression (Fig. 4). Subsequently, a global, unbiased assessment of signaling differences was determined by RNA-seq transcriptome analysis of γδTCR and γδ–αβTCR thymocytes in CD1d–OP9DL4 epithelial cultures with and without sulfatide addition. These data clearly showed that stimulation of γδ–αβTCR, relative to that of γδTCR, induced greater regulated expression of a multiplicity of genes in the DN4 compartment, many of which are specifically associated with T cell stimulation, adhesion control, chemotaxis, signaling, and cellular metabolism (Fig. 6).

Increasing evidence supports a model of gene network–driven lymphocyte lineage diversification preceding antigen receptor expression (64, 65). Although TCR signaling might contribute to γδT lineage fate, gene network drivers per se are a dominant component. Along these lines, we find by PCA that DP10.7 γδTCR manifest small differences in gene expression in the presence or absence of sulfatide in stromal cultures, relative to the γδ–αβTCR chimera. It is important to view the cellular results presented herein strictly as an indicator of the signaling capacity of the given receptors, and not as a study of developmental pathways of γδT cells per se, since aside from hints of preferential chimeric receptor deletion with sulfatide addition, evidence for ligand-directed progression was not unequivocal within the OP9–DL4 stromal system for this γδTCR.

γδT lineage cells are the first to exit the thymus, having already acquired effector function and been programmed to populate different anatomical epithelial locales linked to their Vγ usage (reviewed in ref. 66). These cells demonstrate ligand recognition straddling both innate and cognate immune spaces. A majority of γδTCR ligands are self-derived and stress-induced in lymphoid and nonlymphoid cells, including epithelial cells. For example, CD1 molecules as well as T10/T22 have prominent display in human and mouse thymus, respectively, with CD1d shared between species (67). The recognition by TCRs, even αβTCRs, of ligands expressed at high copy numbers does not require mechanosensing (20). Therefore, if γδT cell ligands are densely arrayed constitutively or upon up-regulation by cellular perturbations involving stress responses, including inflammation, then ligand multivalency per se is adequate to stimulate T cell signaling. Reduction in ligand density or attenuation of T cell signaling severely curtails class IIb T22/T10 reactive transgenic KN6 γδTCR-expressing thymocyte fate in favor of αβ (43, 68–70). Insofar as the CβFG loop fosters DN progression and is essential to mediate effective negative selection (57, 58), the absence of demonstrable positive or negative selection of γδTCR-expressing thymocytes further fits with our observations. If γδ thymocytes were similar to αβ thymocytes, then high copy number of ligands such as with T22 binding receptors would stimulate deletion (71). Given that the γδTCR is tuned to respond to strong signals by virtue of ligand multiplicity, the results in the KN6 studies follow logically (43).

It is noteworthy that lymphoid progenitors begin to rearrange TCRγ-, δ-, and β, but not α-genes at DN2. Those DN3 thymocytes simultaneously expressing γ- and δ-proteins array surface γδTCRs, whereas those expressing β-proteins paired with invariant pTα express pre-TCRs. By contrast, the α-gene is rearranged and expressed only subsequently at the DP thymocyte stage. The sequential αβ T lineage-tuning pathway dependent on mechanosensing at both pre-TCR and αβTCR stages is critical to permit αβT cells to distinguish between foreign versus self-peptides bound to identical MHC molecules arrayed on the same target cell where the representation of the relevant foreign ligand may be on the order of 1 relative to 10,000 self-peptides. The γδTCR need not mediate this level of specificity and digital sensitivity and hence requires no sequential selection steps for repertoire formation. Instead γδTCRs imbue γδT cells with the capacity to focus on their critical sentinel function of nonpeptide recognition in designated barrier tissues and internal organs employing innate and adaptive triggering mechanisms. Given that γδTCR ligands are distinct from and often more plentiful than conventional class I and class II MHC molecules, CD8αβ or CD4 coreceptors are not required, although a subset of γδT cells express CD8αα, CD8αβ, or CD4 (16, 66). While other receptor systems deploy sequence-related functional variants in different tissues (e.g., voltage-gated sodium channels) (72), the T lineage avails itself of a particular implementation. Its receptors comprise ligand-binding subunit variants differing in their capacity to amplify bioforces and thus to modulate triggering of cellular activation using a comparable set of signaling (CD3) subunits. Other distinctions between TCRαβ and TCRγδ, including their connecting peptides and transmembrane segments (56), in addition to their ectodomains, might further nuance signaling differences.

To mount a robust response, signals are processed at many levels, including integrated input from multiple types of receptors, feedback loops within the cell, and communication among populations of cells (73). From a signaling and systems biology perspective, high acuity αβT cells are able to interpret rare input from a handful of peptides to drive a digital output. Here we stress the individual αβTCR as being critical to the αβT cell signal processing, amplifying the signal at the point of input and utilizing the αβ constant domains and transmembrane elements to aid signal interpretation (28). Gated detection is a second strategy that places a window around a signal input and isolates it from noise outside of this window. Force may serve to “gate” the αβTCR signal input. Bond strengthening and conformational change require energy that is sustained for the cognate αβTCR–pMHC interaction (29), while weak interactions are gated out as noise. A third strategy, feedback, appears to be operating at the level of the αβTCR through both an active myosin-based transport that sustains optimal force (21, 74) and a passive method consequent to local membrane stiffness that buttresses this critical force. These mechanisms not only maximize bond lifetime but permit repeated conformational changes that foster a fourth strategy for signal processing, namely resonant detection.

A consequence of such a digital output is a loss of the ability to spread the response over a larger range of input conditions, i.e., in an integrative or analog mode. γδT cells may benefit from reduced sensitivity at the individual receptor level by retaining the ability to integrate signal input across multiple γδTCRs on that T cell. Integration across plentiful individual signals could be advantageous in sensing a gradient or threshold, in the presence of higher ligand concentrations representative of common target antigens for γδT cells. Similar analog αβTCR–pMHC interactions may be involved in positive selection in the thymus, homeostatic T cell proliferation in the periphery, or antiviral responses to high-density ligands on infected cells. Thus, while αβT cells may exploit both modalities, γδT cells appear to be designed exclusively for analog signaling. The implications of this distinction and further analysis of their molecular mechanisms may have translational impact in areas involving adoptive cellular therapies as well as vaccine design.

## Materials and Methods

### Choice of TCR Structures for MD Simulations.

For the WT TCRαβ, N15 TCRαβ Protein Data Bank (PDB), 1NFD (33) and JM22 TCRαβ, 1OGA (75), were used. PDB 1NFD corresponds to the N15 TCRαβ used in experiments. JM22 TCRαβ PDB 1OGA has a bound pMHC and is currently the highest in resolution available (1.40 Å). For the WT TCRγδ, we used 9C2, PDB 4LFH (76). For the Vγδ–Cαβ hybrid, we used DP10.7 TCRγδ, PDB 4MNH (31) and replaced the Cαβ part with those from PDB 1NFD, to match the construct with the one used in experiments (DP10.7-N15). This was done by aligning TCRs of 1NFD and 4MNH using Modeler (77) and replacing the Cαβ part of 4MNH with that of 1NFD. For Vδ, residues up to L119 were kept, after which was Cα of 1NFD. For Vγ, residues up to P119 were kept, followed by Cβ of 1NFD. To ensure that the replacement of constant domains has minimal impact on the interface with the variable domains, we compared interdomain contacts between the original 4MNH structure and the one with Cαβ from 1NFD, prior to performing simulation. We found that they have very similar hydrogen bonds and nonpolar contacts, using nearly identical sets of residues, which reflect the sequence homology of the constant domains in 4MNH and 1NFD. The Vαβ–Cγδ chimera was built similarly, using the Vαβ domain of PDB 1NFD (up to residue 112 in both α- and β-chains) and the rest from the Cγδ of PDB 4LFH, starting from R120 of the δ-chain and K126 of the γ-chain. Missing loops in structures were built using Modeler, and hydrogen atoms were added in CHARMM (78). None of the missing residues are at the interdomain interface, hence they do not affect our interdomain contact analysis. Disulfide bonds were placed as they appear in respective domains.

### MD Simulation and Analysis.

Simulations were performed using CHARMM (79). Each construct was solvated in a cubic water box of about 98 Å in each dimension, which has boundaries at least 12 Å away from the protein. Sodium and chloride ions were added at about 50 mM concentration to neutralize the system. The simulation system underwent a series of energy minimization procedures (4,000 steps in total), where a set of gradually decreasing harmonic restraints was applied to the protein to remove close contacts and relax the surrounding water molecules and ions. After initial energy minimization, the system was heated from 30 K to 300 K during 100 ps, and equilibrated at 300 K for 200 ps. During heating and equilibration, backbone heavy atoms were harmonically restrained with a spring constant of 5 kcal/mol⋅Å^2^. Pressure was maintained at 1 atm using the constant pressure and temperature thermostat. Harmonic restraint was then reduced to 0.001 kcal/mol⋅Å^2^, applying only to the backbone alpha carbon atoms, and an additional 2-ns dynamics run was performed at 300 K in the constant atom number, volume, and temperature ensemble. Finally, a 300-ns production run was performed without any restraint applied (120 ns for the Vαβ–Cγδ chimera). Cutoff distance for nonbonded interaction was 12 Å. The particle-mesh Ewald summation method was used to account for the long-range electrostatic interactions. The SHAKE algorithm was used to fix the length of the covalent bond between hydrogen and heavy atoms. For integration, a 2-fs time step was used, and coordinates were saved every 20 ps. A periodic boundary condition was applied during the simulation. The domain decomposition module was used for efficient parallelization (79).

Contact analysis was performed as done previously (29, 80). Briefly, a 2.4-Å cutoff distance between a hydrogen atom and hydrogen bond acceptor atoms was used to identify hydrogen bonds. For nonpolar contacts, a 3.0-Å cutoff distance between atoms with the absolute value of partial charge less than 0.3*e* (*e*: charge of an electron) was used. Occupancy of a contact was calculated as the fraction of coordinate frames during 100 to 300 ns (50 to 120 ns for the Vαβ–Cγδ chimera; *SI Appendix*, Fig. S1*E*). For the contact statistics in Fig. 2*A*, 10 36.4-ns windows that overlap 50% (i.e., 100.0 to 136.4 ns, 118.2 to 154.5 ns, etc.) were used and contact occupancies were calculated in each window. Windows were made to overlap to avoid a contact appearing to have a low occupancy if spread between two nonoverlapping windows.

### Single-Molecule Protein Production.

DP10.7 γδTCR (WT) and γδ–αβTCR (chimeric) constructs for SM experiments were produced as previously described (31, 81) except for the insertion of flexible linker and LZ motifs (82) prior to the 3C protease site and 6× His Tag. δ or δ/α were fused to the basic LZ and γ or γ/β were fused with acidic LZ (10). Ectodomain regions end with the heterodimer-forming Cys residue in all constructs. Sequences were confirmed by DNA (Sanger) sequencing. Protein was produced as described (31, 81) with additional anti-LZ purification as described (10, 19, 30, 31, 35, 81, 82). N15αβ was produced as described (4, 82). Biotinylated CD1d, without exogenous ligand and sulfatide bound, and VSV8/K^b^ were produced as described (4, 31, 81). DNA and protein sequences are included in Dataset S2.

### Single-Molecule Tweezers Experiments Tether Geometry and Connectivity and Optical Tweezers Measurements.

The tether geometry and optical tweezers measurements parallel assays performed in refs. 19, 30. Additional details are in *SI Appendix*, *Materials and Methods*.

### Constructs for Cellular Experiments.

DP10.7 γδTCR (WT) and γδ–αβTCR (chimeric) constructs, for cellular and SMSC experiments were cloned from previously reported constructs (31, 81). Viral 2A-linked system sites were inserted between subunits to create single δ-p2a-γ or δ-α-p2a-γ-β constructs for WT or chimeric constructs, respectively, as previously published for γδ and αβ TCR (30, 31, 38, 81). Chimeric constructs incorporate the constant domain, connecting peptide region and TM region of the α- or β-subunit as appropriate. Site-directed mutagenesis was used to create appropriate *Eco*RI and *Not*I restriction sites for insertion into LZRS-IresGFP (addgene) retroviral vectors for use in OP9–DL4 stromal cell cultures (35). DNA and protein sequences are included in Dataset S2.

### Generation of BW5147.3 Cell Lines.

BW5147 cells were cotransduced with plasmids pMIY encoding CD3δγεζ (a gift of the Vignali Laboratory, St. Jude Children’s Research Hospital, Memphis TN) and pMIGII (Addgene) encoding either DP10.7 γδ or γδ–αβ essentially as described in ref. 28. Additional details are in *SI Appendix*, *Materials and Methods*.

### SMSC Assay.

The SMSC assay was carried out essentially as detailed (19). Additional details are in *SI Appendix*, *Materials and Methods*.

### OP9–DL4 Stromal Cell Culture.

OP9–DL4 and OP9–DL4–CD1d stromal cell cultures were performed as described (35). Additional details are in *SI Appendix*, *Materials and Methods*.

### Generation of OP9–DL4–hCD1d.

Since the human D10.7 γδTCR recognizes sulfatide presented by human CD1d, by necessity the OP9–DL4 cells, which express low levels of mouse CD1d (*SI Appendix*, Fig. S5*B*), required modification to express human CD1d. Mouse β2m can associate with human HLA class I heavy chains to generate an expressed heterodimer, but may generate both glycosylation and structural differences that differ from the human β2m-containing heterodimer (83–85). To introduce human β2m and to maximize the likelihood that the human CD1d only associates with human β2m, we generated a single-chain hβ2m–hCD1d construct in pcDNA3.1-zeo for stable expression in OP9–DL4 (*SI Appendix*, Fig. S5 *A* and *B*). Additional details are found in *SI Appendix*, *Materials and Methods*.

### Flow Cytometry and Cell Sorting.

For cell-surface molecule staining, transduced cells were first treated with anti-mouse CD16/CD32 mAbs (2.4 G2) in staining buffer (2% fetal bovine serum and 0.05% sodium azide in phosphate buffered saline) to block FcR binding and then stained with antibodies. Antibodies used in this study are listed below. Zombie Aqua (BioLegend) was used for staining dead cells. Intracellular IL-17 and IFN-γ staining was performed after stimulation with 50 ng/mL PMA, 500 ng/mL ionomycin, and 5 μg/mL Brefeldin A for 4 h. For intracellular staining, cells were fixed with 4% paraformaldehyde and treated with permeabilization buffer (0.1% saponin in staining buffer) and then incubated with the antibodies against intracellular cytokine. Lymphocytes and thymocytes from a 9-wk-old female C57BL/6 (Taconic) mouse were used for the positive control of cytokine expression. Cells were analyzed on an LSR Fortessa (BD Biosciences) as described below. Data were analyzed with FlowJo software (Tree Star). The APC-conjugated DP10.7 TCR tetramer was produced as described (31). For DP10.7 TCR tetramer staining, OP9–DL4, OP9–DL4–MHC KO, and OP9–DL4–CD1d stromal cells were plated at 2.5 × 10^4^ cells followed by overnight incubation. Cells were cultured with or without sulfatide (3 μg/mL) for 2.5 h and then stained by 500 nM tetramer in 10% human serum/staining buffer.

### Transcriptome Analysis.

A total of 2,000 γδTCR- or γδ–αβTCR-transduced GFP+ CD45+ DN3 cells were sorted, plated onto OP9–DL4–CD1d cells, which were plated the day before at 5 × 10^4^ cells in six-well plates and cultured in OP9 medium supplemented with Flt-3, IL-7, and gentamicin with or without sulfatide. After 8 d, 5,000 live DN3 (GFP^+^CD45^+^CD4^−^CD8^−^CD44^−^CD25^+^) cells and DN4 (GFP^+^CD45^+^CD4^−^CD8^−^CD44^−^CD25^−^) cells were sorted (*SI Appendix*, Fig. S8).

For each condition (γδTCR or γδ–αβTCR, DN3, or DN4, plus or minus sulfatide, yielding eight conditions/experiment), 5,000 cells were deposited into 350 µL of TCL lysis buffer (Qiagen) and stored at −80 °C until RNA isolation. Three independent experiments generated 24 libraries. Total RNA was then purified from the stabilized lysates using the ARCTURUS PicoPure RNA isolation kit (Thermo Fisher). Following RNA purification, residual DNA was removed by treatment with the Turbo DNA-Free reagent kit (Thermo Fisher). Total RNA was quantified using the Qubit RNA assay kit (Life Tech) and RNA quality was determined on a bioanalyzer using the RNA Pico kit (Agilent). The NuGen Ovation Human RNA-Seq Multiplex system (NuGen, part 0341) prep kit, was used to target deletion of unwanted high abundance transcripts and ribosomal RNA. More than 100 ng of total RNA was converted into each DNA library following the manufacturer’s protocol without modification. Following library construction, DNA libraries were quantified using the Qubit High Sensitivity DNA kit (Life Tech), and library size was determined using the Bioanalyzer High Sensitivity Chip kit (Agilent). Finally, qPCR was carried out on the libraries using the Universal Library Quantification kit for Illumina (Kapa Biosystems) and run on the 7900 HT Fast qPCR machine (ABI). Libraries passing quality control were diluted to 2 nM in sterile water and then sequenced on the NextSeq500 (Illumina) at a final concentration of 12 pM, following all manufacturer protocols.

### Antibodies.

Antibodies are detailed in *SI Appendix*, *Materials and Methods*.

### Bioinformatic and Statistical Analysis.

The output fastq files were aligned against the Ensembl GRCm38.75 reference genome using STAR aligner (v2.5) (86) and the resultant binary alignment map (BAM)-format files were filtered to retain only primary-aligned reads (samtools view -F 0 × 0100). The read counts were quantified at the exon level using subRead featureCounts (v1.4.4) software (87) and differential expression testing was performed using DESeq2 (v1.6.3) software (88). Individual results were considered to be of significance only if the *p.adj* ≤ 0.1 (q value; multiple-test corrections were performed using the Benjamini–Hochberg procedure). Immune cell signatures were determined using the MSigDB (C7: immunologic signatures) package hosted at the Broad Institute (47).

### Multisequence Alignment.

TCR Cγ and TCR Cβ sequences and equivalent CH1 sequences of selected Ig isotopes were aligned using three-dimensional structural information with PROMALS3D (89). All sequences were obtained from the international ImMunoGeneTics information system (90) but those of ferret TCR Cγ (accession no. XP_012914450.1) and cow TCR Cβ (accession no. AAI42018.1) were obtained from GenBank after BLAST searches with human counterparts.

## Supplementary Material

Supplementary File

Supplementary File

Supplementary File

## Data Availability

Sequencing read and summary files have been deposited in the National Center for Biotechnology Information Gene Expression Omnibus database (ID GSE165297). All other study data are included in the article and/or supporting information.
