# Supplementary material for: Molecular design of the γδT cell receptor ectodomain encodes biologically fit ligand recognition in the absence of mechanosensing
Source: Proc Natl Acad Sci U S A. 2021 Jun 25;118(26):e2023050118. doi: 10.1073/pnas.2023050118 (PMC8256041; doi:10.1073/pnas.2023050118)
Supplement: Supplementary File [file pnas.2023050118.sapp.pdf]

PNAS
www.pnas.org

Supplementary Information for
Molecular design of the γδ T cell receptor ectodomain encodes biologically fit ligand recognition in the absence of mechanosensing

Robert J. Mallis[a,b,c], Jonathan S. Duke-Cohan[a,c], Dibyendu Kumar Das[d], Aoi Akitsu[a,c], Adrienne M. Luoma[e], Debasis Banik[d], Hannah M. Stephens[d], Paul W. Tetteh[a.c], Caitlin D. Castro[e], Sophie Krahnke[e], Rebecca E. Hussey[a], Brian Lawney[f], Kristine N. Brazin[a,c], Pedro A. Reche[g], Wonmuk Hwang[h,I,*], Erin J. Adams[e,*], Matthew J. Lang[d,*] & Ellis L. Reinherz[a,c,*]

[a]Laboratory of Immunobiology, Dana-Farber Cancer Institute, Boston, MA 02115

[b]Departments of Biological Chemistry & Molecular Pharmacology and Dermatology, Harvard Medical School, Boston, MA 02115

[c]Department of Medical Oncology, Dana-Farber Cancer Institute and Department of Medicine, Harvard Medical School, Boston, MA 02115

[d]Department of Chemical & Biomolecular Engineering and Molecular Physiology & Biophysics, Vanderbilt University, Nashville, TN 37235

[e]Department of Biochemistry and Molecular Biology, University of Chicago, Chicago IL 60637

[f]Department of Biostatistics, Harvard T.H. Chan School of Public Health, Boston, MA 02115

[g]Department of Immunology, Faculty of Medicine, Universidad Complutense de Madrid, Madrid Spain, 28040 SP

[h]Departments of Biomedical Engineering, Materials Science & Engineering, and Physics & Astronomy, Texas A&M University, College Station, TX 77843

[i]School of Computational Sciences, Korea Institute for Advanced Study, Seoul 02455, Korea

*Address correspondence to Erin J. Adams, University of Chicago, 929 E. 57th St, Chicago, IL 60637, 773-834-9816, ejadams@uchicago.edu; Wonmuk Hwang, 3120 TAMU, College Station, TX 77843, 979-458-0178; Matthew J. Lang, Vanderbilt University, PMB 351604, 2301 Vanderbilt, Nashville, TN 35235, 617-875-7493, matt.lang@vanderbilt.edu; or

Ellis L. Reinherz, Dana-Farber Cancer Institute, 35 Binney St. Room JF518, Boston, MA 02115; 617-632-3412; ellis_reinherz@dfci.harvard.edu

This PDF file includes:

Supplementary Methods
Figures S1 to S8
Legends for Datasets S1 to S2
SI References

Other supplementary materials for this manuscript include the following:

Datasets S1 to S2

Materials and Methods

Single molecule tweezers experiments tether geometry and connectivity and optical tweezers measurements

The tether geometry parallels assays performed in (1, 2). Tether connectivity consisted of 1% PEG-biotin functionalization on the coverslip, streptavidin and CD1d.

Carboxyl-polystyrene beads (1 μm diameter, Polysciences) were covalently reacted with an amine terminated 3520-bp double-stranded DNA strand containing covalently bound half-antibody 2H11 that binds to the TCR construct paired LZ regions. A 10-μl flow cell is prepared using double-sided sticky tape and tethers formed through successive washes of CD1d, then TCR-bound beads, followed by an exchange wash to remove unbound tethers.

Tethers were found by eye, centered by moving the coverslip surface using a piezo stage and an automated bead centering Labview program and pulled by moving the coverglass surface relative to a fixed trap with a ramp profile to a defined trap-bead separation where the stage was held fixed at the desired force until rupture. The trap stiffness was typically ~0.2pN/nm. Bead trap separations were typically 100nm or less, well in the linear range of the trap stiffness. Bead position was antialias filtered at 1.5 kHz and recorded at 3 kHz. After rupture, position sensing was mapped and stiffness calibrated for that particular bead.

Generation of BW5147.3 Cell lines

Cells were cultured in high glucose DMEM medium (Sigma-Aldrich) supplemented with 10% fetal bovine serum (FBS, Sigma-Aldrich), 100 U/ml penicillin, 100 U/ml streptomycin (P/S, Life Technologies), 55 μM 2-mercaptoethanol, 2mM L-glutamine (Corning), 400 μg/ml Geneticin (Gibco), 400 μg/ml Hygromycin B (Invitrogen). The cell lines were generated by transfection of CD3- and TCR-containing plasmids into Phoenix-Eco packaging cells (ATCC) for separate retrovirus production. The viral supernatants were harvested and used to retrovirally transduce BW5147 cells to incorporate CD3 and TCR genes in that order selecting on the YFP and then GFP marker within the respective plasmids. Cells were routinely FACS sorted to maintain equivalent surface TCR levels selecting on anti-CD3ε 145-2C11 APC staining.

SMSC Assay

Tethers were constructed from half anti-biotin antibody functionalized DNA (3500 bp) attached to anti-digoxygenin-coated polystyrene beads (1.0 μm diameter, Spherotech, Inc.) at one end and C-terminally biotinylated CD1d-sulfatide at the other. Beads were washed twice with PBST (PBS + 0.02% Tween-20) and diluted 200-fold with 5 mg/ml bovine serum albumen (BSA, Sigma-Aldrich) in colorless DMEM (Sigma-Aldrich) for bond-lifetime measurements. Cells were washed with colorless DMEM and resuspended to 2 x 10^6 cells/ml. Cell suspension (20 μl) was transferred to the flow chamber (at 37°C and 5% CO_2) and cells were allowed to bind to the coverslip. After 30 min incubation, the coverslip surface was blocked with an additional wash with 5 mg/ml BSA in colorless DMEM. After 10 min further incubation 20 μl pMHC-DNA bead slurry was added to the same chamber. Tether functionalized beads were trapped, calibrated and brought towards the cell to actively form a stable tether. Tethers were then loaded by translating the cell relative to the fixed trap. In some cases, beads were also calibrated following measurement to assure the stability of the system.

Single cell activation requirements (SCAR) assay

The method for cell activation follows the procedure described in Feng, et al. (3). Streptavidin coated polystyrene beads (1.09 μm, Spherotech Inc.) at 0.1% w/v were combined with varying dilutions of biotinylated CD1d-sulfatide and rotated for 1 h to achieve the targeted ligand density on the bead surface. Beads were washed in PBST, and the surface blocked with 5 mg mL^{-1} biotin-BSA to prevent non-specific binding. The interface concentration was determined as described (3). Cells at a concentration of $2x10^6$ cells mL^{-1} were incubated with Ca^{2+} sensitive Quest Rhod-4 dye in colorless DMEM media with 3% FBS for 40 min to allow cellular uptake of dye. The cells were washed with colorless DMEM and loaded into a flow cell comprised of a microscope slide with a KOH etched coverslip. The slide was incubated for 1 h at 37°C. After incubation, a blocking buffer containing 5 mg mL^{-1} BSA in colorless DMEM media was added to the flow cell and

incubated for 10 min to block the surface. The CD1d-sulfatide bead solution was then added to the flow chamber.

The slide was loaded into the optical tweezers and fluorescence instrument. A ligand-coated bead was captured using the 1,064 nm trapping laser calibrated and translated to the surface of the T cell to facilitate bond formation between the TCR and CD1d-sulfatide molecule. After a baseline fluorescence image was collected, a shear force was applied by moving the piezoelectric stage nanometer distances tangent to the surface of the T cell. Fluorescence images were captured at 10 s intervals over a 10 min window.

OP9-DL4 Stromal Cell Culture

Fetal liver progenitor cells from *Rag2*$^{-/-}$ mice were cultured for 6 days, and then transduced with γδTCR, γδ-αβTCR, or N15αβ TCR cDNA constructs in LZRS-IresGFP (addgene) followed by overnight incubation. 2,000 transduced GFP^{+} CD45^{+} CD4^{-} CD8^{-} CD44^{-} CD25^{+} (DN3) cells were sorted and plated onto adherent OP9-DL4 or OP9-DL4-CD1d cells, passaged the day before at 5×10^4 cells in OP9 medium (αMEM, 15% FBS, and penicillin/streptomycin) supplemented with Flt-3 (5 ng/ml; R&D Systems), IL-7 (1 ng/mL; Peprotech), and gentamicin (5 μg/ml; Sigma-Aldrich) with or without sulfatides [3 μg/ml; brain, porcine, 24:1 (36%); 24:0 (24%); Avanti Polar Lipids] onto 6 well tissue culture plates. The cell cultures were maintained for 8 days at 37°C. Statistical analysis of stromal cell culture development and CD3 population levels was by linear regression analysis compensating for experimental variability to determine significance (p) using the R software package.

Generation of OP9-DL4-hCD1d

For construction of single chain CD1d-β2M, human CD1d was amplified from pcDNA3.1-neo-CD1D by PCR (Forward: gctcGGATCCGAAGTCCCGCAAAGGCTTTTC;

Reverse: cgaGCGGCCGCgTCACAGGAC) to generate a *Bam*HI-*Not*I flanked CD1d with the start codon and signal peptide removed and then recloned into pcDNA3.1-zeo digested with *Bam*HI/*Not*I. The following oligonucleotides were annealed to each other (Forward: cttGGTACCTCAGGTGGTGGCGGTTCAGGCGGAGGTGGCTCTGGCGGTGGCGGATCGGGTGGCGGTGGCTCGGGATCCacta; reverse: tagtGGATCCCGAGCCACCGCCACCCGATCCGCCACCGCCAGAGCCACCTCCGCCTGAACCGCCACCACCTGAGGTACCaag) to generate 5'-*Kpn*I-$(G_4S)_4$-linker-*Bam*HI-3' that was then ligated into *Kpn*I/BamHI-digested pcDNA3.1-CD1d-zeo. Human β2m was amplified from IMAGE consortium clone:5502428 (in pCMV-SPORT6; Forward: acttAAGCTTgccgagATGTCTCGCTCCGTG; reverse: cttcGGTACCCATGTCTCGATCCCACTTAAC) to generate a 5'-*Hind*III-β2m-*Kpn*I insert maintaining the start codon and signal sequence of human β2m with deletion of the intrinsic stop codon. The β2m was then inserted into the *Hind*III/*Kpn*I-digested pcDNA3.1-zeo-linker-CD1d to generate the full single chain construct (Fig. S5A).

Antibodies

CD117 2B8 APC, BD Pharmingen; CD117 2B8 APC, BD Pharmingen; Sca-1 D7 FITC, eBioscience; CD8β 53-5.8 PE, BioLegend; CD8α 53-6.7 Brilliant Violet 605, BioLegend; CD8α 53-6.7 PerCP/Cy5.5, BioLegend; CD4 RM4-5 Pacific Blue, BioLegend; CD4 RM4-5 Brilliant Violet 605, BioLegend; CD25 PC61.5 PE-Cy7, Invitrogen; CD44 IM7 APC-Cy7, BD Pharmingen; CD45 30-F11 APC, BioLegend; CD45 30-F11 Brilliant Violet 711, BioLegend; CD3ε 145-2C11 APC, BioLegend; IL-17 TC11-18H10.1 Pacific Blue, BioLegend; IFN-γ XMG1.2 APC, BioLegend; TCRγ/δGL3 PE, BioLegend; Paired leucine zipper 2H11 (4).

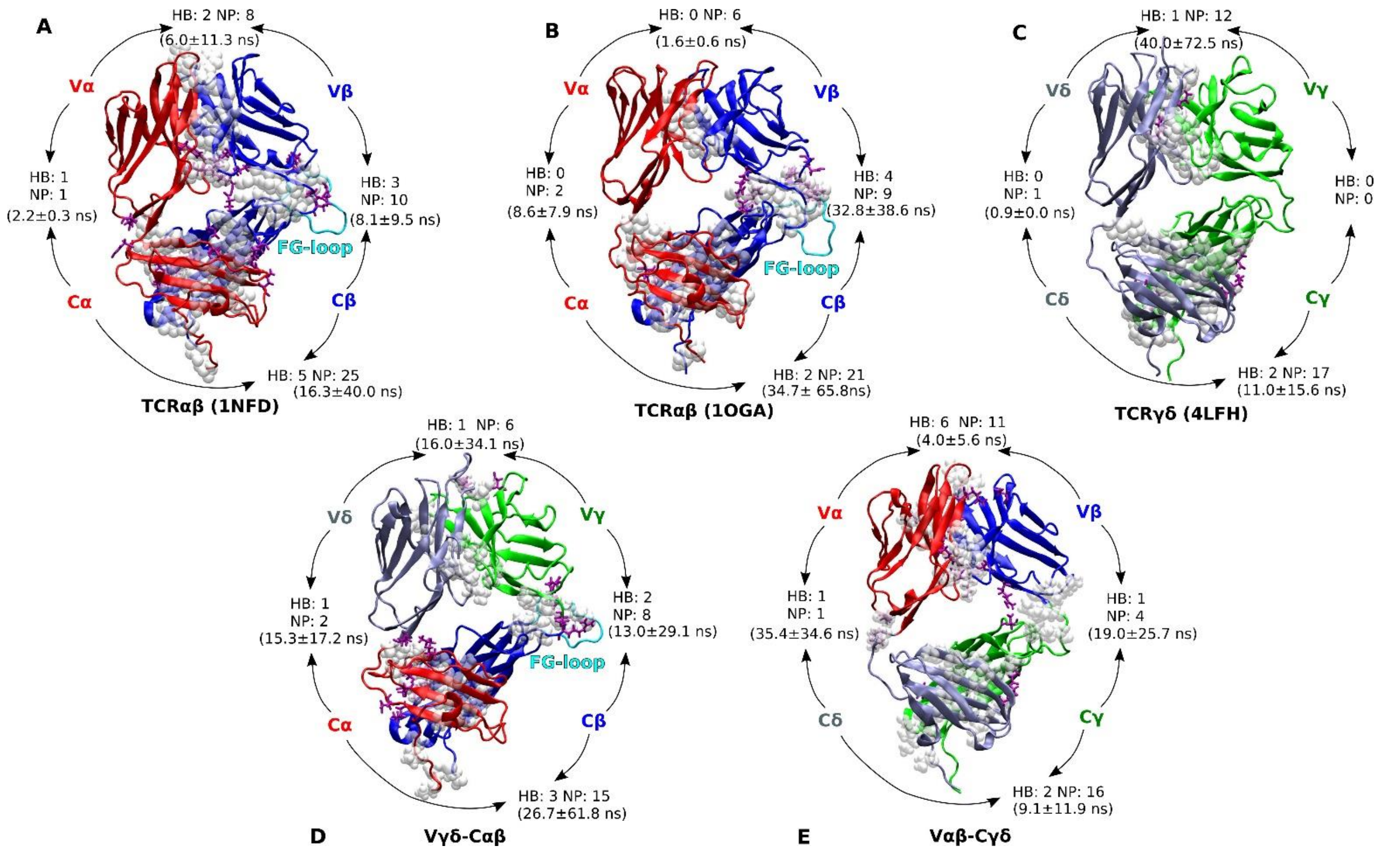

Figure S1. Contact Maps of αβ-, γδ-, and chimeric TCRs. A.-D. Structural representation of interdomain contacts delineated by molecular dynamics simulations (Fig. 2). Ribbon structure models are decorated with white spheres showing the residues participating in interdomain nonpolar contacts (NP). Violet sticks show residues participating in polar contacts (HB). **A.** TCRαβ; N15αβ **B.** TCRαβ; JM22αβ **C.** TCRγδ; 9C2γδ **D.** TCRγδ-αβ. **E.** TCRαβ-γδ. Contacts with greater than 80% occupancy were counted during the 100-300ns MD simulation interval (50-120ns for panel E). Average contact lifetime ± standard deviation for these contacts, shown in parentheses, are much shorter than simulation time.

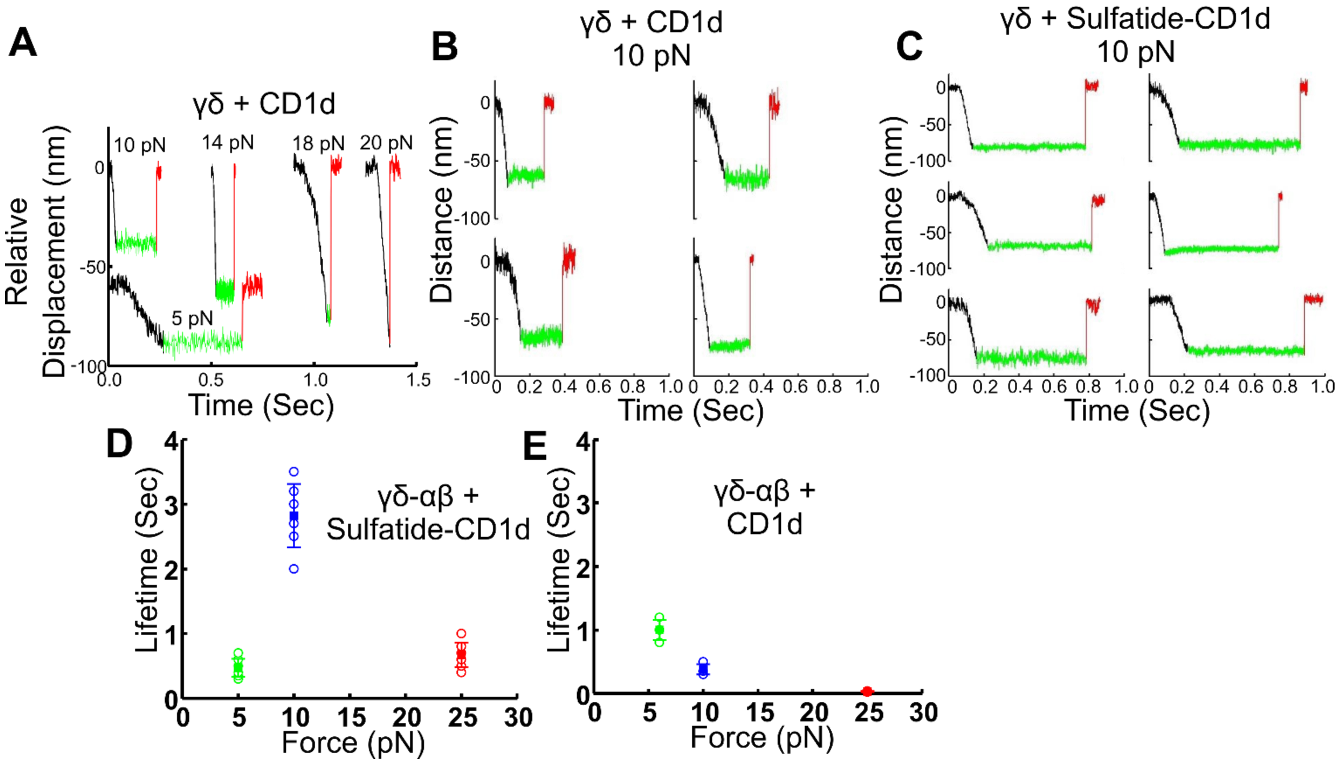

Figure S2. Representative data on γδ and γδ-αβ SM assays. A. Representative traces depicting relative displacement vs. time are shown for γδ + CD1d over a range of forces. **B-C.** Traces comparing γδ binding without (B) and with (C) sulfatide at 10 pN. **D-E.** γδ-αβ + Sulfatide-CD1d (D) or γδ-αβ + CD1d (E) lifetime vs. force plots showing individual SM measurements as open circles with mean values as a filled square. Error bars represent standard deviation boundaries for measurements from data in Figure 3F at 5, 10 and 25 pN for γδ-αβ + Sulfatide-CD1d and Figure 3G at 6, 10 and 25 pN for γδ-αβ + CD1d lacking sulfatide.

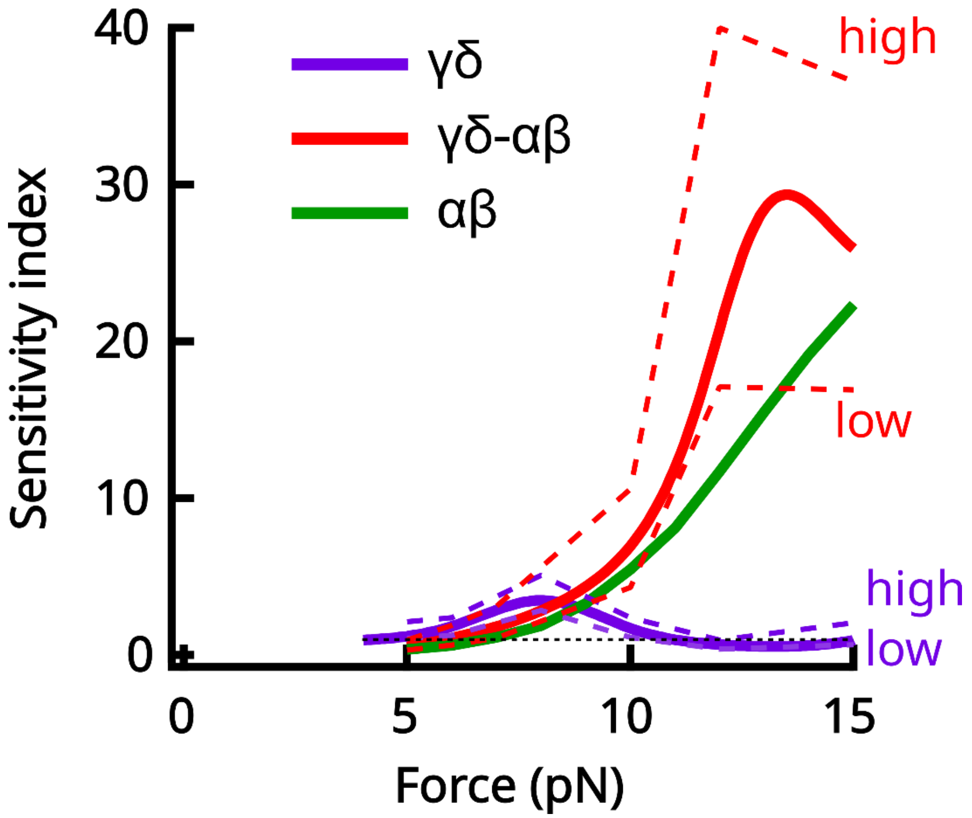

Figure S3. Sensitivity index (SI) comparison of WT and Chimeric DP10.7. Sensitivity index (SI) for DP10.7γδ (purple), DP10.7γδ-αβTCR (red) or N15αβTCR (green) as a function of force. Note that plots are stopped at 15pN as binding to the less specific CD1d or SEV9-K^b tends to 0s lifetime. Plots were constructed from spline interpolations to the data in Figure 3E for γδ or γδ-αβTCR to generate a continuous curve. The SI comparison is generated by evaluating the following equation at every force point [SI = (bond lifetime with Sulfatide-CD1d)/(bond lifetime with CD1d)]. Estimates to high and low boundaries of the SI evaluation were determined from the standard error of the mean (SEM) of the original data in Fig. 3E. Boundaries are shown as dashed lines in their respective colors. High and low boundaries were calculated at every discrete force point found in the data. When necessary, mean and SEM at a particular force value was estimated by interpolating the errors from neighboring points. The "high boundary" was estimated assuming the upper confidence limit (mean lifetime + SEM) for the numerator

and lower confidence limit (mean lifetime – SEM) for denominator. Similarly, the “low boundary” was estimated assuming the lower confidence limit of the numerator and upper confidence limit of the denominator. A dotted line is also shown at SI=1. Data for N15αβ [SI = (bond lifetime with VSV8-K^b)/(bond lifetime with SEV9-K^b)] interaction is from (1).

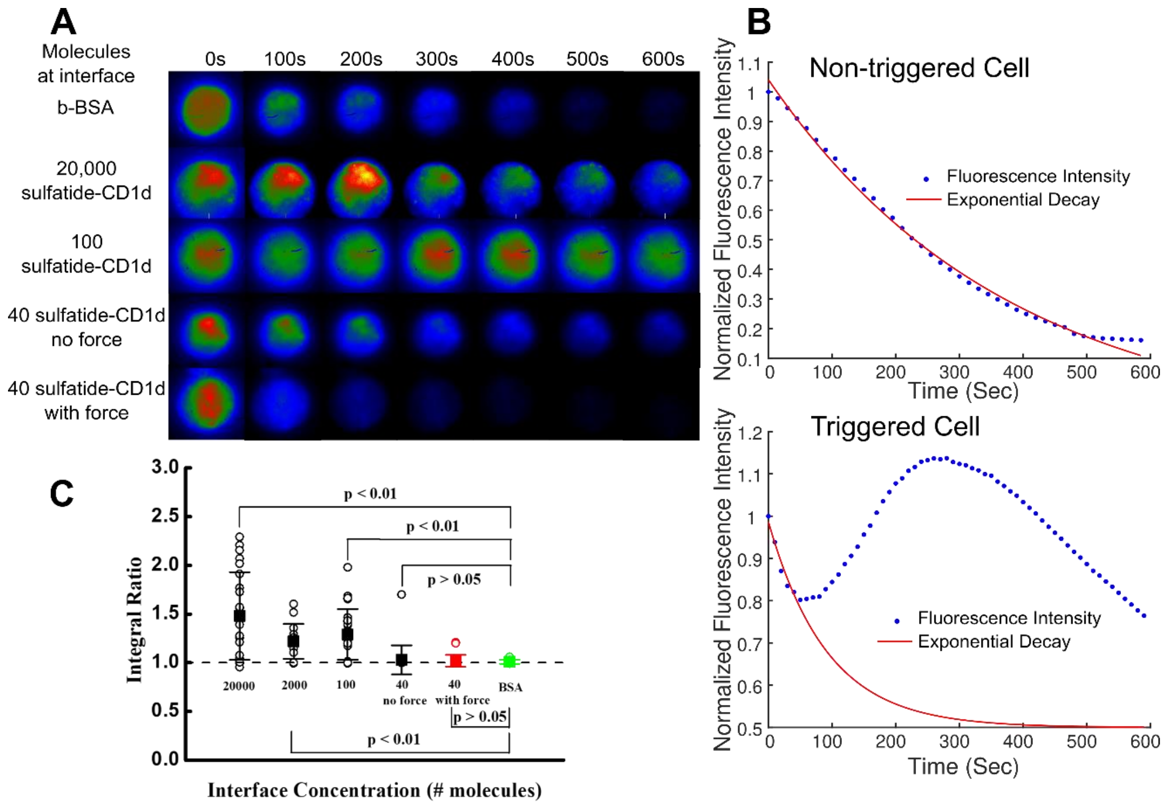

Figure S4. Effect of force on triggering individual γδ T cells with single cell activation requirements (SCAR) assay. A. Beads were coated with sulfatide-CD1d, interfacial sulfatide-CD1d concentration counted and cells triggered as detailed in Supplementary Methods and as in (3). Beads were trapped with optical tweezers and brought to the surface of the cell to facilitate binding. Triggering was observed with no force at $2x10^4$ (18 of 24), $2x10^3$ (11 of 15), and 100 (18 of 27) molecules at the interface. With 40 molecules at the interface, however, no triggering was observed in the absence of force (1 of 21). Subsequently, cells were tested with 40 molecules at the interface with applied forces ranging from 5-25 pN and triggering was still not observed, indicating the absence of mechanosensing function (2 of 20). Beads coated with BSA were used as a negative control. Time in seconds is given for each cell being monitored and intracellular calcium monitored as described (3). **B.** Normalized fluorescence intensity was collected for each cell over a 10-minute window. Cells that do not trigger (top panel) exhibit

photobleaching of the Ca^{2+} sensitive dye (blue trace) that fits an exponential decay profile (red trace). A successfully triggered cell (bottom panel) shows a rise (blue trace), deviating from a typical exponential decay profile (red trace) expected of a non-triggered cell. **C.** Quantitation of cell triggering using the integral ratio of data/non-triggering decay for each interfacial concentration. An integral ratio of 1 indicates an exponential decay consistent with non-triggered cells, while integral ratio >1 indicates triggering. As detailed in panel B, for activated cells an exponential fit to the initial decay given typical fit values consistent with non-triggered cells provided an estimate for the numerator in the ratio. For activating cells, deviation from an exponential decay profile produces an integral ratio greater than 1. Open circles represent raw data, with mean and SD overlayed. P values were determined using one-way ANOVA.

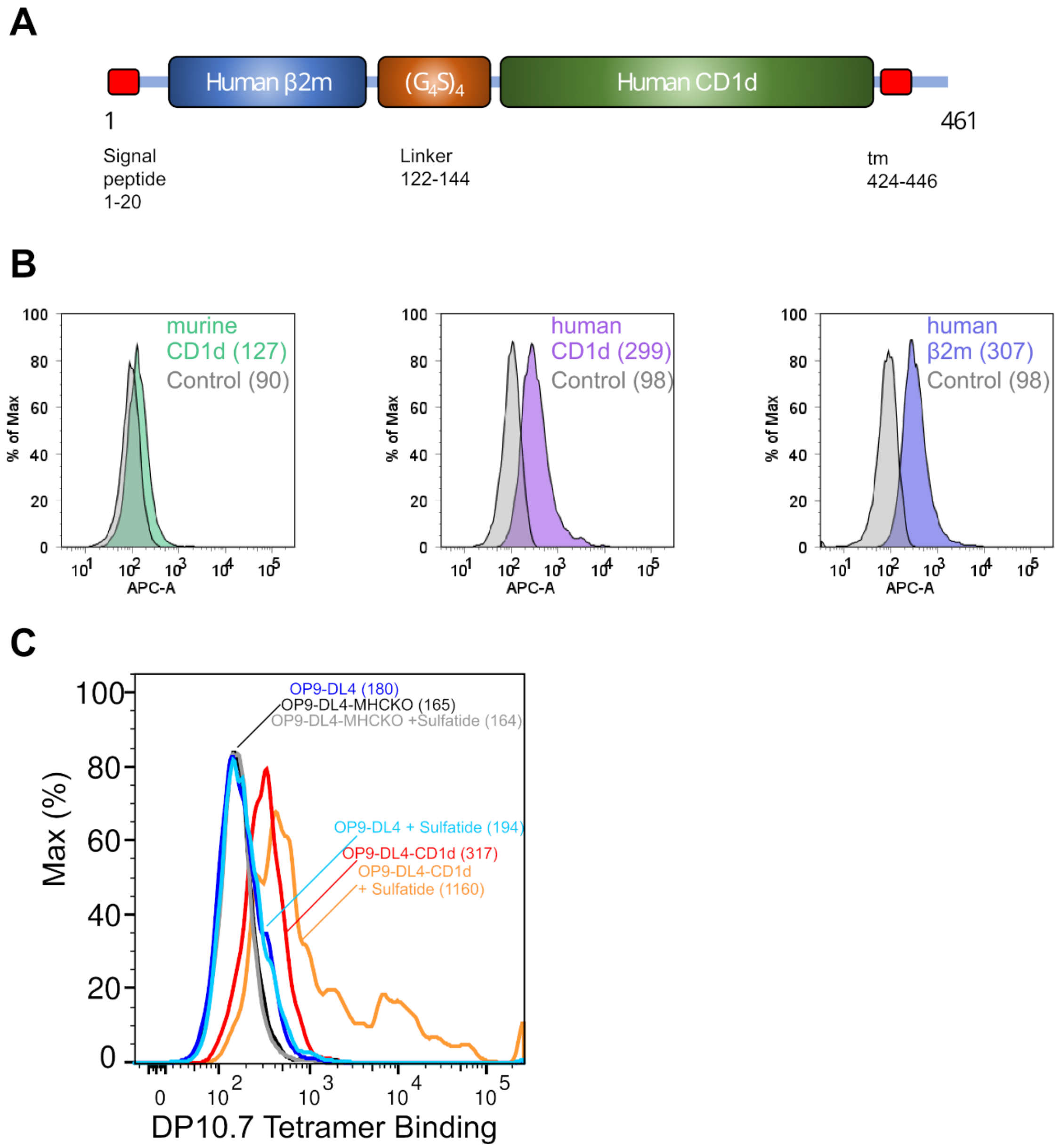

Figure S5. OP9-DL4-CD1d stromal cell line generation and validation. A. Domain structure of single chain human β2m-human CD1d used to generate stably-transfected OP9-DL4-hCD1d. **B**. Stably transfected OP9-DL4-CD1d cells express mouse CD1d, human CD1d and human β2m. Colored traces are stained with the indicated antibody specificity. Grey traces are stained with appropriate isotype control mAb, rat IgG2b-APC for mouse CD1d and mouse IgG2b-APC for human CD1d and β2m. Geometric median is indicated for each profile in parentheses. **C.** DP10.7 tetramer binding

analysis of OP9-DL4 stromal cell lines. FACS plot of OP9-DL4, OP9-DL4-CD1d, or OP9-DL4 modified to remove expression of MHC Class I molecules including murine CD1d (OP9-DL4-MHCKO) (2) to show DP10γδ TCR tetramer binding levels in the absence and presence of sulfatide as indicated. Geometric median for each profile is indicated in parentheses.

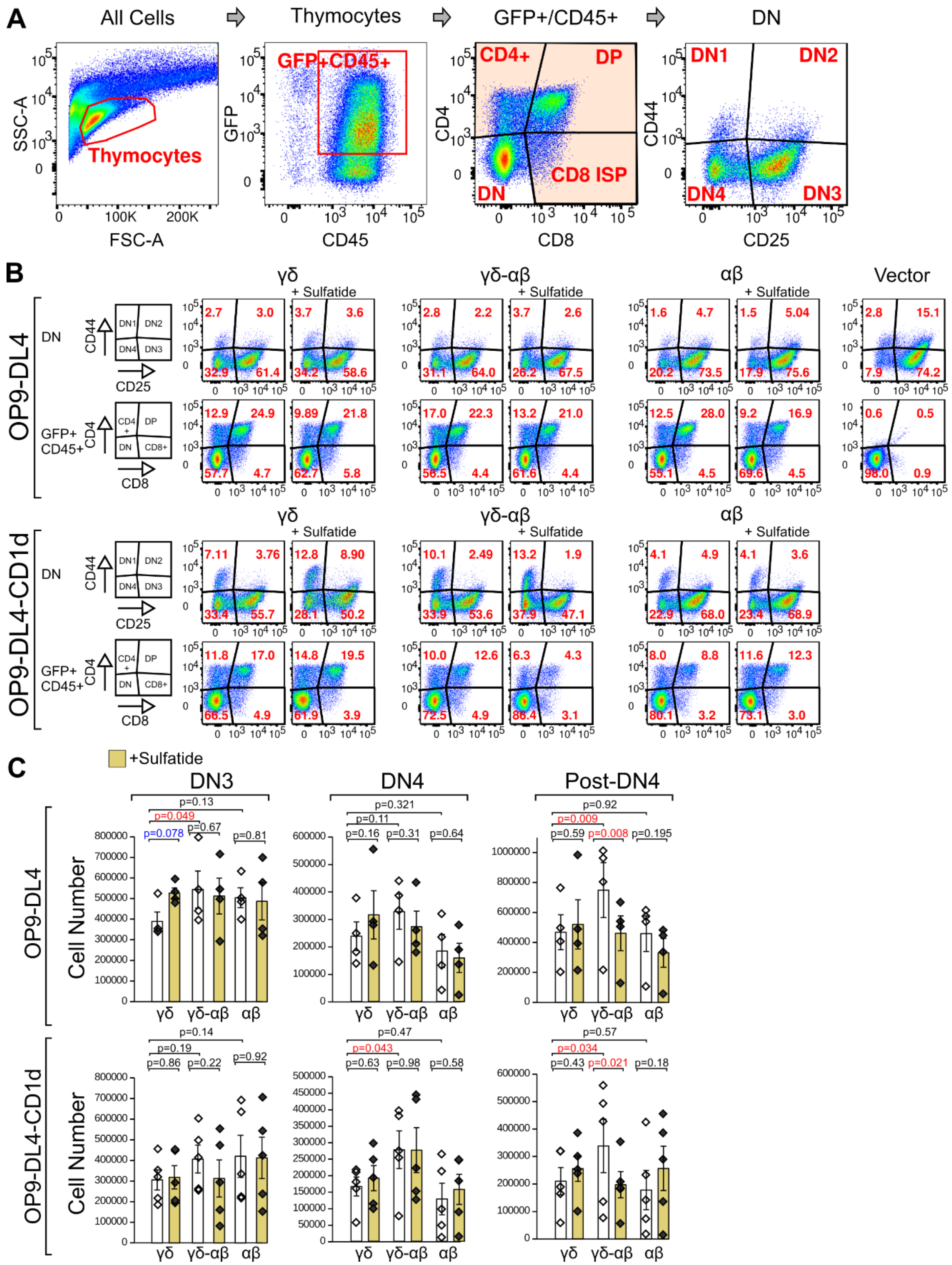

Figure S6. Developmental analysis of transduced thymocytes on stromal cell cultures with added sulfatide. A. Gating strategy. All cells were gated with FSC-A and

SSC-A to isolate thymocytes then GFP^{+}CD45^{+} to select transfected thymocytes. CD4^{+}CD8^{+} = DP; CD4^{-}CD8^{-} = DN; CD25 and CD44 staining is used to determine DN3 (CD44^{-}CD25^{+}) or DN4 (CD44^{-}CD25^{-}) within the DN subset. Analysis of γδTCR-transduced thymocytes cultured with OP9-DL4 cells for 8 days is shown. Post-DN4 is defined as the orange shaded region comprising CD8 ISP, CD4+ and DP thymocytes. **B.** Representative FACS analysis of DP10.7 TCRγδ (γδ) or DP10.7γδ-αβ (γδ-αβ) transduced thymocytes cultured for 8 days in the presence or absence of sulfatide in co-culture with parental OP9-DL4 stromal cells or OP9-DL4 cells stably transfected with human CD1d (OP9-DL4-CD1d). Analysis is of DN subsets or CD4 and CD8 status of GFP+CD45+ thymocytes gated as shown in A following culture of γδTCR (γδ), γδ-αβTCR (γδ-αβ), N15αβ (αβ) or vector transduced thymocytes with the indicated stromal cell lines. **C.** Statistical analysis of four (OP9-DL4) or five (OP9-DL4-CD1d) independent experiments as represented in B. White bars are from cultures treated with DMSO vehicle only, yellow are sulfatide treated. Significance (p value) was determined by linear regression analysis. Significant ($p<0.1$) or highly significant differences ($p<0.05$) are highlighted in blue and red, respectively.

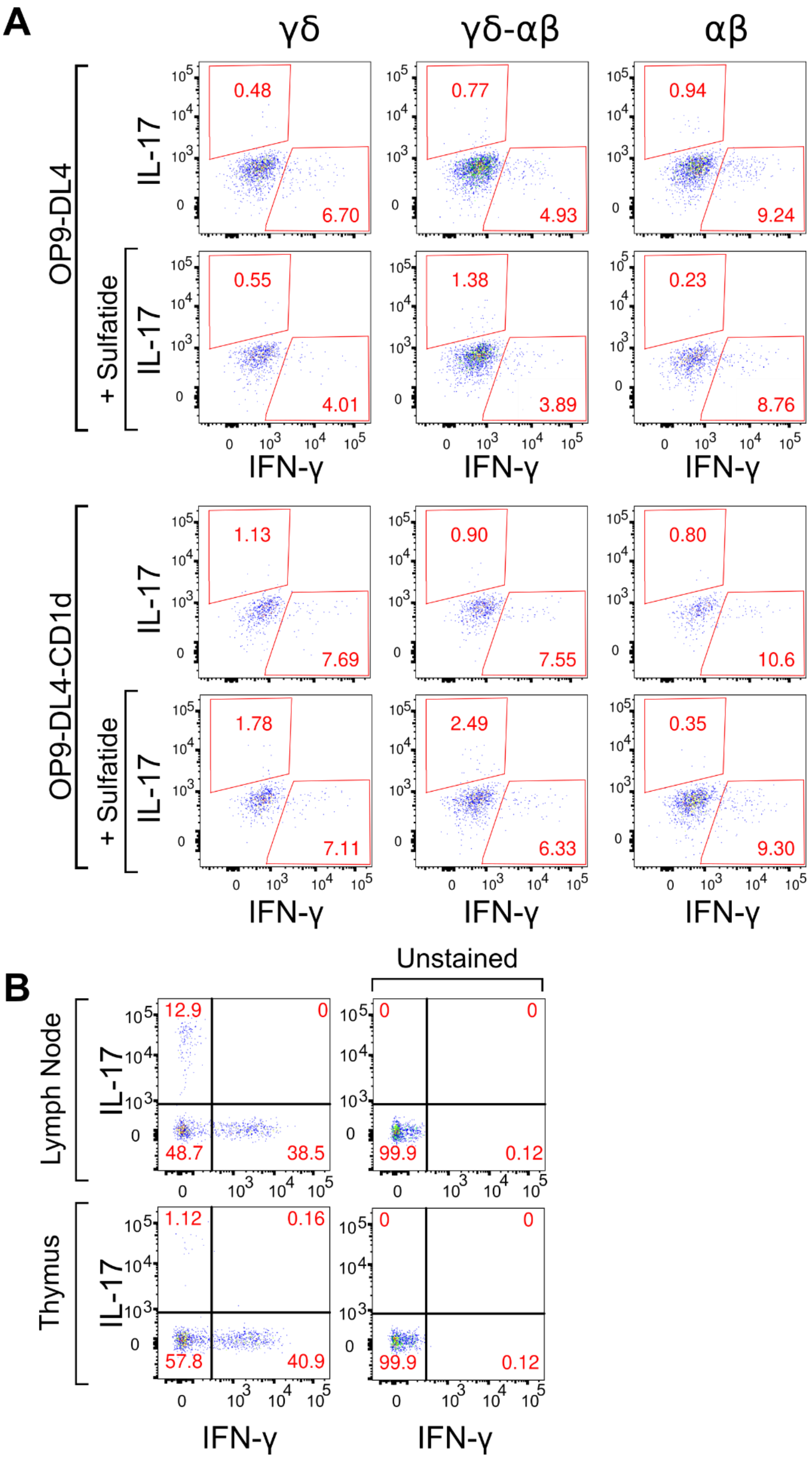
A
γδ
γδ-αβ
αβ
OP9-DL4
+ Sulfatide
OP9-DL4-CD1d
IL-17
IFN-γ
0.48
6.70
0.77
4.93
0.94
9.24
0.55
4.01
1.38
3.89
0.23
8.76
1.13
7.69
0.90
7.55
0.80
10.6
1.78
7.11
2.49
6.33
0.35
9.30
B
Unstained
Lymph Node
Thymus
12.9
0
48.7
38.5
0
0
99.9
0.12
1.12
0.16
57.8
40.9
0
0
99.9
0.12

Figure S7. IL-17 and IFN-γ production in γδTCR-, γδ-αβTCR-, and N15αβ-expressing thymocytes. A. IL-17 and IFN-γ production in γδTCR- (γδ), γδ-αβTCR-(γδ-αβ), and N15αβ- (αβ) transduced cells cultured with OP9-DL4 or OP9-DL4-CD1d cells with or without sulfatide for 8 days followed by Ionomycin (500ng/ml) and PMA (50ng/ml) stimulation as described in main text are shown. Cells are gated as $GFP^{+}CD45^{+}CD4^{-}CD8^{-}$ (DN). **B.** IL-17 and IFN-γ production of γδT cells in lymphocytes (Lymph Node) and thymocytes (Thymus) from a 9 week old female C57BL/6 mouse are shown (B6, left) as FACS staining controls. Cells without staining by IL-17 and IFN-γ mAbs are shown (Unstained, right). All cells are gated as γδ TCR^{+} cells with mAb GL3.

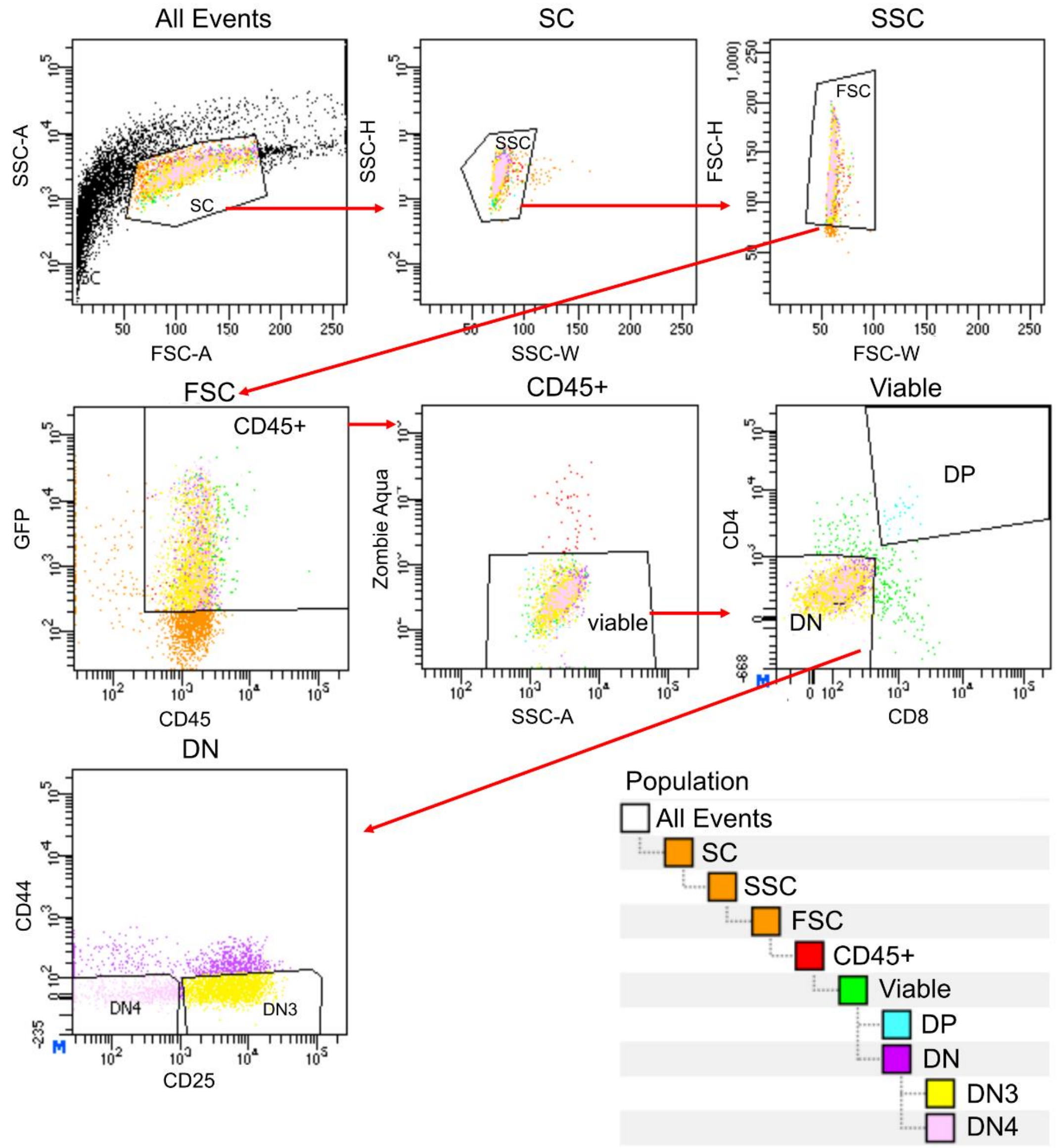

Figure S8. Gating scheme for sort of DN3 and DN4 thymocytes targeted for transcriptomic analysis. Lymphocytes were gated based on forward (FSC) and side scatter (SSC) profiles. Within the lymphocyte population, singlets, $CD45^+GFP^+$, viable cells, and $CD4^-CD8^-$ (DN) were gated and DN3 and DN4 cells were sorted based on $CD44^-CD25^+$ and $CD44^-CD25^-$, respectively. GFP was used for the marker of successfully γδ- and γδ-αβ-transfected cells.

Dataset S1. (Excel Spreadsheet) Transcriptomic analysis supporting information. 1-All Data-mean-sem. Gene transcript expression levels (mean counts ± sem); for all libraries, n = 3, except for γδ-αβTCR.DN3.no-stim and γδ-αβTCR.DN4.no-stim where n = 2. Data not normalized. **2-DN3>DN4drivers.** Top 100 genes contributing to Principal Component Analysis for DN3 to DN4 transition. **3-DN3>DN4-ranked.** Fold-change (log2) for DN3 to DN4 transition for all gene transcripts. **4-DN3 γδTCR-stim.** Fold-change (log2) in γδTCR-transduced Rag2-/- DN3 cells in response to sulfatides presented by human CD1d. **5-DN3 γδ-αβTCR-stim.** Fold-change (log2) in γδ-αβTCR-transduced Rag2-/- DN3 cells in response to sulfatides presented by human CD1d. **6-DN4 γδTCR-stim.** Fold-change (log2) in γδTCR-transduced Rag2-/- DN4 cells in response to sulfatides presented by human CD1d. **7-DN4 γδ-αβTCR-stim.** Fold-change (log2) in γδ-αβTCR-transduced Rag2-/- DN4 cells in response to sulfatides presented by human CD1d.

Dataset S2. (Excel Spreadsheet) DNA and amino acid sequences for proteins used in SM, SMSC and cellular experiments.

SI References

1. D. K. Das *et al.*, Force-dependent transition in the T-cell receptor beta-subunit allosterically regulates peptide discrimination and pMHC bond lifetime. *Proc Natl Acad Sci USA* **112**, 1517-1522 (2015).
2. D. K. Das *et al.*, PreTCRs leverage Vβ CDRs and hydrophobic patch in mechanosensing thymic self-ligands. *J Biol Chem* **291**, 25292-25305 (2016).
3. Y. Feng *et al.*, Mechanosensing drives acuity of αβ T cell recognition. *Proc Natl Acad Sci USA* **114**, E8204-E8213 (2017).
4. J. Liu *et al.*, Crystallization of a deglycosylated T cell receptor (TCR) complexed with an anti-TCR Fab fragment. *J Biol Chem* **271**, 33639-33646 (1996).
